# Imprecise intron losses are less frequent than precise intron losses but are not rare in plants

**DOI:** 10.1186/s13062-015-0056-7

**Published:** 2015-05-27

**Authors:** Ming-Yue Ma, Tao Zhu, Xue-Nan Li, Xin-Ran Lan, Heng-Yuan Liu, Yu-Fei Yang, Deng-Ke Niu

**Affiliations:** MOE Key Laboratory for Biodiversity Science and Ecological Engineering, College of Life Sciences, Beijing Normal University, Beijing, 100875 China; Beijing Computing Center, Beijing, 10094 China; Present address: Institute of Genetics & Developmental Biology, Chinese Academy of Sciences, Beijing, 100101 China

**Keywords:** Intron loss, De-intronization, De-exonization, Insertion, Deletion, *Solanum*, *Arabidopsis thaliana*

## Abstract

**Abstract:**

In this study, we identified 19 intron losses, including 11 precise intron losses (PILs), six imprecise intron losses (IILs), one de-exonization, and one exon deletion in tomato and potato, and 17 IILs in *Arabidopsis thaliana*. Comparative analysis of related genomes confirmed that all of the IILs have been fixed during evolution. Consistent with previous studies, our results indicate that PILs are a major type of intron loss. However, at least in plants, IILs are unlikely to be as rare as previously reported.

**Reviewers:**

This article was reviewed by Jun Yu and Zhang Zhang. For complete reviews, see the Reviewers’ Reports section.

**Electronic supplementary material:**

The online version of this article (doi:10.1186/s13062-015-0056-7) contains supplementary material, which is available to authorized users.

## Findings

Theoretically, five different types of molecular events can inactivate introns or cause the deletion of an intron from a gene, thereby contributing to a decrease in intron abundance (Additional file 1). The first type of event is precise intron loss (PIL); in this case, intron losses do not affect the integrity of flanking exons. The second type of event is imprecise intron loss (IIL), which is accompanied by the insertion and/or deletion (indel) of nucleotides into/from flanking exons. The third type of event is termed de-intronization; in this case, sequences are not deleted from the genome, but rather an intronic sequence is converted into an exonic sequence by mutations that deactivate splicing signals. The fourth type of event is termed de-exonization, which is the conversion of an internal exon into an internal portion of an intron by mutations that deactivate splicing signals. This process leads to the fusion of an exon and its flanking two introns, which creates a larger intron and therefore decreases the intron number. Finally, the deletion of an internal exon also results in the fusion of two neighboring introns and consequently decreases the intron number by one. In this paper, we used the term “intron loss” in a broad sense to include all five of the above types of intron variations. Almost all previously observed intron losses are PILs; IILs and other types of intron losses appear only rarely [1-15]. There are three possibilities for the observed patterns. The first possibility is that they occur at quite different frequencies. For example, if intron losses are mediated by mRNA molecules, then all intron losses should be PILs [16]. The second possibility is that intron losses that change coding sequences have essentially been eliminated by purifying selection. The third possibility is that there is a methodological bias toward the identification of PILs. It is possible for intron losses to introduce indels into coding sequences and therefore significantly reduce the similarities between flanking coding sequences and their orthologous regions. To be confident in identifying cases of intron loss, researchers generally discard poorly aligned regions [9-11].

### IILs are less frequent than PILs but are not rare in *Solanum* or *Arabidopsis thaliana*

**Figure 1:**
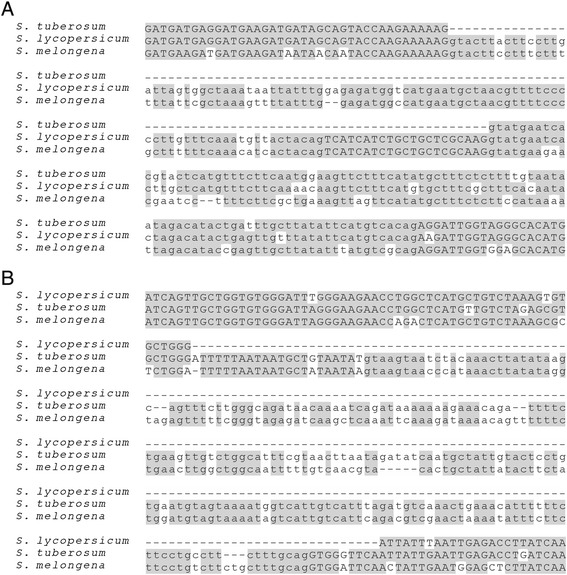
Two examples of imprecise intron losses. **(A)** The *S. tuberosum* gene *PGSC0003DMG400000276* lost both an intron and a 21-bp-long downstream exon. The downstream intron was intact and did not lose any nucleotides; its splicing was supported by > 10 RNA-Seq reads. The splicing of the target intron in *S. lycopersicum* was also supported by > 10 RNA-Seq reads and by EST asmbl_392.tomatov23pasa_pasa4. The assembly of the variation site in *S. tuberosum* was supported by nine different Whole Genome Shotgun (WGS) reads. **(B)** The *S. lycopersicum* gene *Solyc04g007270.2* lost a 204-bp-long intron, a 22-bp-long segment from an upstream exon and a 9-bp-long segment from a downstream exon. This deletion occurred in the 3'-UTR and did not inactivate the gene, as supported by >10 RNA-Seq reads. The successful splicing of the target intron in *S. tuberosum* was supported by > 10 RNA-Seq reads. The assembly of the variation site in *S. lycopersicum* was supported by > 10 WGS reads. For details of the RNA-Seq and WGS reads and other intron losses, see Additional file 2 and Additional file 3.

As the genomes of tomatoes (*Solanum lycopersicum*) and potatoes (*Solanum tuberosum*) diverge by less than 10% [17], we obtained reliable alignments for most of their orthologs. By surveying intron-exon structural changes in tomato and potato, we found 11 cases of PIL and six cases of IIL (Figure 1 and Additional file 2).

The species *A. thaliana* diverged from its relative *Arabidopsis lyrata* less than ten million years ago [18]. In comparing the genomes of these species, we found 17 IILs in *A. thaliana* (Additional file 3). A close examination of 114 cases of intron loss from *A. thaliana* that were reported in a previous study [19] revealed that 104 of these cases, which occurred in 98 genes, were PILs, two were IILs, and eight lacked support due to insufficient numbers of RNA-Seq reads. The two cases of IILs were included in our 17-case dataset of IILs.

### Nearly all ILs have been fixed during evolution

Transcriptome data showed that all of the variant genes in our study were still actively expressed (Additional files 2 and 3). A close examination of these genes did not reveal any premature stop codons that were introduced by intron loss mutations. The IIL genes are unlikely to be pseudogenized. Indels caused by IILs in coding sequences are more likely to be selected against. It is possible that the IILs that we observed were recent events that would soon be eliminated. This possibility could be excluded if the variations that we observed in one species were also found to exist in another. For this reason, we investigated whether tomato’s wild relative, *Solanum pimpinellifolium*, shares intron variations with tomato. In *S. lycopersicum*, there are five IILs and five PILs. We surveyed the orthologous genes in the genome of *S. pimpinellifolium* and found all ten of the variations. Therefore, all of the intron losses that we observed in *S. lycopersicum* have been fixed during evolution.

Taking advantage of the availability of genome sequences corresponding to multiple *A. thaliana* lines, we also tested whether the intron variations that were observed in *A. thaliana* have been fixed during evolution. By surveying 17 IIL genes in 180 lines of *A. thaliana* from Sweden [20], we found that all 17 cases of IILs had been fixed. Similarly, by surveying 104 PILs in 180 lines of *A. thaliana*, we found that 101 cases of PIL have been fixed during evolution and only three PILs remained polymorphic, in genes *AT1G48420*, *AT3G23080*, and *AT4G00350*. However, the allele frequencies of these three PILs were high: 97.7% for *AT1G48420*, 36.1% for *AT3G23080* and 97.2% for *AT4G00350*.

IILs are expected to be under negative selection because of the indels that they cause in coding sequences; therefore, a majority of IILs might be eliminated. Nevertheless, the fixed IILs were still found to comprise an appreciable proportion of intron losses in *Solanum* and *Arabidopsis*.

### The relative frequencies of IILs were underestimated when comparing distantly related genomes

The practice of filtering unreliable alignments may have led to underestimates of the frequencies of IILs in previous studies. If this were the case, a higher proportion of IILs than PILs would have been undetectable when we compared each of the two *Solanum* genomes with a distantly related species versus when we compared the two *Solanum* genomes to each other. To test this possibility, we surveyed for the presence of *Solanum* IL genes in the genome of a rice, *Oryza sativa*, that diverged from *Solanum* 163 million years ago [18]. Among the 11 PIL genes and six IIL genes that we surveyed, we found *O. sativa* orthologs for all 11 PIL genes and only two IIL genes. In principle, when a very low identity is observed between two aligned sequences, it indicates that either a large number of mutations accumulated after divergence or that a low-quality alignment was produced. To maintain accuracy, these alignments are generally discarded. We calculated the identities of the coding sequence alignments of the sites flanking the intron losses. For each intron loss, 45-bp-long regions of coding sequence (not including gaps in the alignments) corresponding to positions on each side of the loss were included in the calculation. Using the first quartile of the identities of all aligned coding sequences that were generated between *Solanum* and *O. sativa* (0.53) as the threshold to filter unreliable alignments, one IIL and two PILs in *Solanum* were discarded. In summary, only one of the six IILs that occurred in *Solanum* could be detected when comparing its genome to rice. In contrast, nine of the 11 PILs could be detected using the same method. This difference is statistically significant (χ^2^ test, *P* = 0.03). Similar analyses have been carried out on the introns that have been lost from the genome of *A. thaliana*. Rice and *A. thaliana* also diverged 163 million years ago [18]. Among 17 IIL genes and 98 PIL genes that were found in *A. thaliana*, we found four IILs and 47 PILs when comparing its genome against rice. Thus, a lower frequency of IILs than PILs was observed in *A. thaliana* when the reference genome was *O. sativa* (23.5% *vs.* 45.1%, respectively).

Similar to intron losses, the majority of previous studies on intron gains have been restricted to highly conserved orthologous genes. Among these genes, very few or no intron gains were found in humans, mice, and *Arabidopsis thaliana* [19,21-23]. This is in stark contrast to a study that specifically explored intron gains by evaluating segmental duplications, in which tens of intron gains had been revealed in each of these three species [24]. In that study, intron gains that were accompanied by insertions and/or deletions of coding sequences were not excluded.

Identifying the true relative frequencies of PILs and IILs presents a dilemma: accurate IIL to PIL ratios can only be obtained when recently diverged genomes are compared. However, recent divergence means that only a limited amount of time has elapsed to enable the accumulation of intron loss variations. For these reasons, it would be helpful in the future to extend the current study to additional eukaryotic lineages.

### De-exonization and exon deletion in *Solanum*

We identified one case of de-exonization, one case of exon deletion, two cases of intronization, and one case of exonization in tomato and potato (Additional file 2). In the tomato gene *Solyc09g016940.2*, an internal exon and the 5′ splicing signal of its downstream intron were both lost. In the potato gene *PGSC0003DMG400004043*, an internal exon has been converted into an internal region of a larger intron. In addition to GT-AG boundaries, there are many cis-acting sequence elements and trans-acting factors that facilitate intron recognition and splicing [25]; therefore, it is reasonable to hypothesize that some intron variations do not involve changes to these boundaries.

## Reviewers’ comments

### Reviewer 1: Jun Yu, Beijing Institute of Genomics, Chinese Academy of Sciences

Ma et al. reports an initial look into precisely how intron loss has happened within a particular plant species, where two genome sequences – one domesticated and another wild – are available, and found 19 intron losses, which are supported by transcription evidence. They also took an addition look on the Arabidopsis genome, inspired by their finding from the Solanum species. Different from intron gain, intron loss should be rather rare event as purifying selection always prevents its loss-of-function effect, such as what may happen to IILs. In addition, the form of intron losses in a context of gene structure is of curiosity also, where functional consequences are complex for different forms, such as PILs *vs.* IILs; the latter may have more severe loss-of-function effect than the former that would not change protein coding sequences in theory. The results from Ma et al. are consistent to this speculation. A bit of concern is Figure 4, where results from diverse species were plotted into a trend that is not supported by adequate evidence across enough data from multiple species.

Authors’ response: *We have streamlined our manuscript to conform to the format of Discovery Notes at the request of the Biology Direct Editorial Team. Figure 4 has been deleted*.

### Minor revision

The manuscript needs some further editing and some of the examples are listed as follows:

1. The comma in the title should be eliminated.
  Authors’ response: *We have revised the title.*
2. In Table 3, “number of” should be removed.
  Authors’ response: *This table has been deleted to better streamline our manuscript.*
3. Remove the sentence mentioning the average number of introns per gene since it is neither a good estimate nor relevant to the manuscript.
  Authors’ response: *This sentence has been deleted.*
4. Change “IILs: not much less frequent than PILs in Solanum” to “IIL is (or IILs are) not much…”
  Authors’ response: *This problem has been corrected.*
5. Replace “focused” with “enriched” in “Similar to intron losses, most previous explorations of intron gains were also focused on highly conserved orthologous genes”
  Authors’ response: *This has been corrected*.
  Quality of written English: Needs some language corrections before being published
  Authors’ response: *The language of this manuscript has been edited by a professional language-editing service.*

### Reviewer 2: Zhang Zhang, Beijing Institute of Genomics, Chinese Academy of Sciences

The manuscript by Ma et al. presented comprehensive investigations on intron loss by comparing multiple plant genome sequences, including tomato, potato, Arabidopsis and rice. Based on considerate filtration and exclusions of questionable data, the manuscript concluded that precise intron losses are the major type of intron loss and imprecise intron losses are not so rare as previously reported.

Although I am not an expert in this field, the manuscript is well-written and provides solid results. However, one of my major concerns is why plant species are used for studying intron loss and how about human, monkey, chimp, etc., if also used. As mentioned, it is due to low divergence (e.g., <10% between tomato and potato), but I feel it might be better to provide more background on a variety of species. Accordingly, the related concern is the title “Imprecise intron losses are less frequent than precise intron losses, but not so rare”. If it is unexplored or does not hold true in other species, it would be safe to add “in plants” in the title.

Authors’ response: *We added “in plants” to the title of the revised manuscript and will monitor imprecise intron losses of other lineages.*

Minor comments

1. The y-axis title in Figure 4 should be consistent with the word used in the main text. Also, how’s the correlation between PIL% and divergence time in different species. I think it is positively correlated. If available, plot together in Figure 4 and estimate the loss rate.
  Authors’ response: *This figure has been deleted in the course of streamlining our manuscript at the request of the Biology Direct Editorial Team.*
2. In Abstract, “enable us explore” should be “enables us to explore”
  Authors’ response: *This sentence has been deleted to better streamline our manuscript.*

## Additional files

Additional file 1: **Illustration of different types of intron loss.**

Additional file 2: **Details of intron variations in potato and tomato.** A complete list of supplementary materials, methods, and figures is provided in this file.

Additional file 3: **Details of intron variations in** ***Arabidopsis thaliana.*** A complete list of supplementary materials, methods, and figures is given in this file.

## Abbreviations

BLAST: Basic Local Alignment Search Tool
bp: Base pair
IIL: Imprecise intron loss
IL: Intron-lost
PIL: Precise intron loss
WGS: Whole Genome Shotgun
