# Supplementary material for: Imprecise intron losses are less frequent than precise intron losses but are not rare in plants
Source: Biol Direct. 2015 May 27;10:24. doi: 10.1186/s13062-015-0056-7 (PMC4443532; doi:10.1186/s13062-015-0056-7)
Supplement: Supplementary file 1 — Illustration of different types of intron loss. [file 13062_2015_56_MOESM1_ESM.docx]

Additional File 1

**Imprecise intron losses are less frequent than precise intron losses but are not rare in plants**

Ming-Yue Ma, Tao Zhu, Xue-Nan Li, Xin-Ran Lan, Heng-Yuan Liu, Yu-Fei Yang, and Deng-Ke Niu


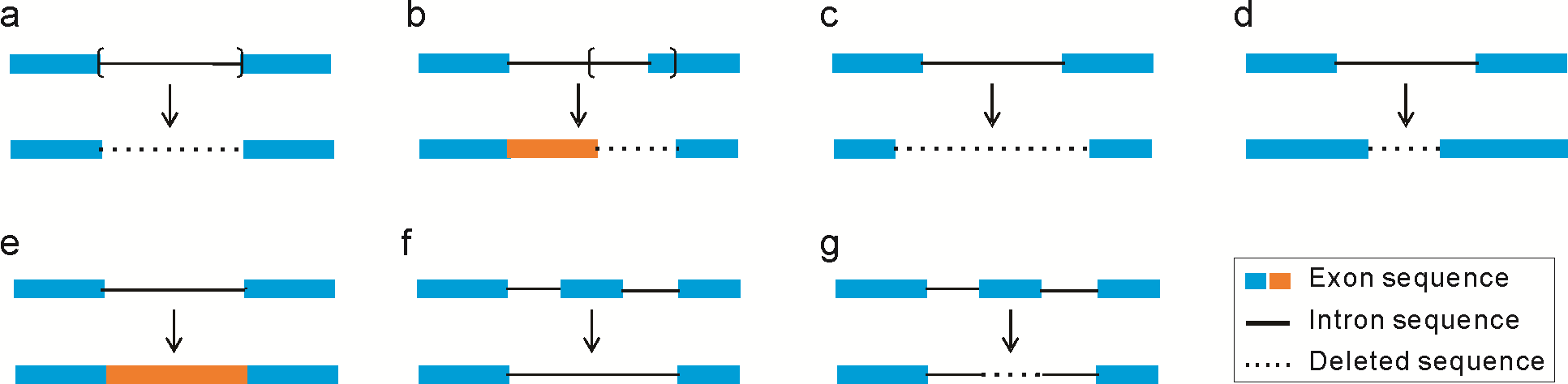


**Figure S1. Illustration of different types of intron loss.** (a) Precise intron loss; (b-d) Imprecise intron loss; (c) De-intronization; (d) De-exonization; (e) Internal exon deletion.
