# Supplementary material for: Imprecise intron losses are less frequent than precise intron losses but are not rare in plants
Source: Biol Direct. 2015 May 27;10:24. doi: 10.1186/s13062-015-0056-7 (PMC4443532; doi:10.1186/s13062-015-0056-7)
Supplement: Supplementary file 2 — Details of intron variations in potato and tomato. A complete list of supplementary materials, methods, and figures is provided in this file. [file 13062_2015_56_MOESM2_ESM.docx]

Additional File 2

**Imprecise intron losses are less frequent than precise intron losses but are not rare in plants**

Ming-Yue Ma, Tao Zhu, Xue-Nan Li, Xin-Ran Lan, Heng-Yuan Liu, Yu-Fei Yang, and Deng-Ke Niu

**Materials and methods**

We downloaded the genome sequences and annotation files of potato (*S. tuberosum*) (PGSC_DM_v3), tomato (*S. lycopersicum*) (ITAG2.3), wild tomato (*S. pimpinellifolium*) (version 1), and tobacco (*N. benthamiana*) (version 0.4.4) [1] from SGN (Sol Genomics Network) [2], those of pepper (*C. annuum* L.) (Zunla-1) from the Pepper Genome Database (release 2.0) [3], and those of *A. thaliana* Col-0 (TAIR release 10), *Glycine max* (JGI Glyma 1.0), *Mimulus guttatus* (JGI gene annotation v1.1 of assembly v1.0), *O. sativa* (MSU Release 7.0), and *V. vinifera* (March 2010 12X assembly and annotation from Genoscope) from Phytozome (version 9.1) [4]. The scaffold sequences of eggplant (*S. melongena*) were downloaded from NCBI (SME_r2.5.1) [5]. The SAR files of the genomes of *S. tuberosum* (SRP007439) and *S. lycopersicum* (ERP003675) and the transcriptomes of *S. tuberosum* (SRP006384 and SRP022916), *S. lycopersicum* (SRP015739 and SRP018993), *C. annuum* L. (SRP019256), *A. thaliana* (SRP009136 and SRP010096), *N. benthamiana* (SRP018508), and *V. vinifera* (SRP014438) were retrieved from the Sequence Read Archive of NCBI [6]. We first filtered the sequences of interest by discarding genes with obvious annotation errors, such as those that did not have coding sequences that were comprised of multiples of three nucleotides or those that appeared to conflict with their protein sequences. Introns that comprised mostly undefined bases (Ns) were also discarded. For genes with multiple splice isoforms, the longest mRNA was retained for analysis.

Using the best reciprocal BLAST hits, orthologous proteins between *S. tuberosum* and *S. lycopersicum* were detected with a threshold E value of < 10^−10^. Additionally, orthologous genes were also identified using SynMap [7]. Overlapping results that were produced by these two methods, which included 14,080 pairs, were regarded as true orthologs and were used for further analysis.

ClustalW and MUSCLE were independently employed to align orthologous genes; their default parameters were used [8, 9]. Results that were consistent were used for to survey intron variability. By consulting the annotations of the orthologous genes, we found 1,056 intron presence-absence variation sites in 912 pairs of orthologous genes. Among these sites, 11 introns contained long stretches of undefined bases (Ns): they were likely to be assembly errors and were therefore discarded. There were two indels that accounted for > 50% of the full coding sequence and therefore should be regarded as gene indels rather than intron indels; they were not included in our analysis. For cases in which multiple indels were observed in close proximity to sites of intron variability, it was difficult to distinguish assembly error, alignment error and multiple mutational events. For this reason, six variation sites were discarded. Among the remaining sites, there are 14 cases of putative precise intron gain or loss, 38 cases of putative imprecise intron gain or loss, 657 cases of putative (de-) intronization, and 328 cases of putative (de-) exonization or indels of internal exon sequences.

To distinguish between intron losses and gains, we examined the absence or presence of introns at the above 52 sites among eight outgroup genomes (Figure S1). The orthologous genes of the outgroup species were identified using the best reciprocal BLAST hits (E value of < 10^−10^). The orthologous sequences in eggplants were manually annotated with references to the annotations of the orthologous genes in potatoes and tomatoes. Referring to previous results that have indicated that intron losses generally outnumber intron gains [10-13], we used standard parsimony to identify intron losses. In total, in *S. lycopersicum* and *S. tuberosum*, we obtained 11 putative cases of PIL and 19 putative cases of IIL (Table S1).


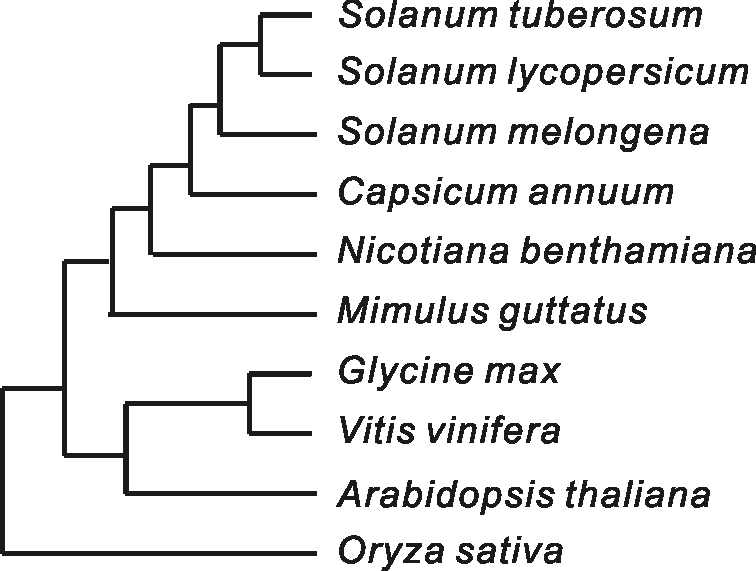


**Figure S1. Phylogenetic tree used to identify intron losses in *S. lycopersicum* and *S. tuberosum*.** The tree was adapted from Phytozome (version 9.1) [4] and Särkinen et al. [14]. Its branches were not scaled according to substitution rates.

PIL can be simply defined as the exact loss of an entire intron. However, for operational purposes, we defined it with two additional criteria: 1) in a species that has not lost a specific intron, the said intron was successfully spliced out of at least one of the abundant mRNA isoforms; and 2) in a species that has lost a specific intron, the intron-lost (IL) gene is still actively expressed. The first criterion ensures that the lost sequence is an authentic intron, rather than an exonic segment that is mis-annotated as an intron. The second criterion excludes intron losses that inactivate IL genes. For studies that focus on the molecular mechanisms of intron loss, the second criterion might not be necessary. However, as the present study aimed to reveal the mutational forces that shape gene structure during evolution, the active expression of IL genes is required. Similarly, IILs were also defined by the above two criteria.

We mapped RNA-Seq reads to reference genomes using TopHat version 2.0.5 with its default parameters [15].

By consulting RNA-Seq data, we found that the vestiges of eight putative cases of IIL could be successfully spliced and should therefore continue to be considered active introns. Therefore, these eight cases were discarded. An additional case of IIL was discarded because the transcriptome data did not support the active expression of the corresponding IL gene. Furthermore, we verified that the lost introns were authentic introns by consulting the transcriptome data of two *Solanum* and outgroup species. In total, 11 cases of putative PIL and six cases of putative IIL were retained.

To avoid artifacts resulting from assembly errors, we tested the qualities of genome assemblies at the sites of the putative intron losses using whole genome shotgun (WGS) reads. The WGS reads were mapped to reference genomes using BWA (alignment via Burrows-Wheeler transformation, version 0.6.1) with its default parameters [16]. For example, the above analysis indicated that the tomato gene *Solyc03g117730.2* had lost its sixth intron. To confirm this finding, we obtained the following evidence. First, seven tomato WGS reads were found to cross the intron-lost position of the tomato gene *Solyc03g117730.2.* Additionally, we tested whether the lost sequence was actually lost or was instead mis-assembled to another position of the genome by mapping the tomato WGS reads to the orthologous gene in potatoes, *PGSC0003DMG400014215*. No reads matched the potato sixth intron, whereas 125 reads matched the flanking region (1 kb at each side). All of the precise and imprecise intron variations passed this filtration step (Data S1).

Most plant genes produce multiple transcripts by alternative splicing. However, select alternative transcripts are targeted by cellular surveillance machinery, such as nonsense-mediated decay, and others may contribute to the proteomic complexity of a cell [17]. A segment that is deleted from a gene might represent an intron loss when considering one transcript, whereas it might be a simple deletion of exon sequence when considering another transcript. Only introns lost from functional transcripts or principal isoforms were relevant to this paper. Therefore, we surveyed the abundance of alternative transcripts with respect to their orthologous genes in each IL gene. In the orthologous genes corresponding to 16 IL genes (11 PILs and 5 IILs), splicing patterns were found to be identical among different alternative transcripts at positions of intron variation. In the orthologous gene that corresponded to the other IIL genes, *Solyc05g025890.1*, the transcripts were different at the position of intron variation. In the potato ortholog of the gene *Solyc05g025890.1*, the target intron was spliced from the most abundant transcript (Data S2). Considering the abundance of the related transcripts, this deletion could be regarded as an intron loss.

**Table S1. Presence and absence of target introns in tomatoes, potatoes and outgroup species.**

| *S. tuberosum* |  | *S. lycopersicum* | | |  | *S. melongena* | | |  | *C. annuum* |  | | *N. benthamiana* | | |  | | *M. guttatus* | |  | | *A. thaliana* | |  | | *V. vinifera* | |  | | *G. max* | |  | | *O. sativa* | |  | |  |  |
| --- | --- | --- | --- | --- | --- | --- | --- | --- | --- | --- | --- | --- | --- | --- | --- | --- | --- | --- | --- | --- | --- | --- | --- | --- | --- | --- | --- | --- | --- | --- | --- | --- | --- | --- | --- | --- | --- | --- | --- |
| **Precise indel** |  |  | | |  |  | | |  |  |  | |  | | |  | |  | |  | |  | |  | |  | |  | |  | |  | |  | |  | |  |  |
| PGSC0003DMG400000530 | + | Solyc02g087120.2 | | | - | DF357785.1 | | | + | 02g003465 | + | | 13513g0004 | | | + | | - | | ? | | AT3G28150 | | - | | 1032519001 | | - | | 19g01510 | | - | | 06g12820 | | ? | |  |  |
| PGSC0003DMG400004708 | - | Solyc12g099540.1 | | | + | DF357741.1 | | | + | 12g000142 | + | | 10747g0021 | | | + | | 001950m.g | | + | | AT3G16630 | | + | | 1017727001 | | + | | 07g37630 | | + | | 05g06280 | | + | |  |  |
| PGSC0003DMG400009895 | - | Solyc04g082170.2 | | | + | - | | | ? | 04g000102 | + | | 46740g0003 | | | + | | 007994m.g | | + | | AT5G42250 | | + | | 1008701001 | | + | | 20g10240 | | + | | 07g42924 | | + | |  |  |
| PGSC0003DMG400010092 | - | Solyc02g089770.2 | | | + | - | | | ? | 00g002881 | + | | 60306g0004 | | | + | | 008662m.g | | ? | | AT5G14700 | | + | | 1036464001 | | + | | 19g00980 | | + | | - | | ? | |  |  |
| PGSC0003DMG400013384 | - | Solyc03g005140.1 | | | + | DF366368.1 | | | + | 06g001914 | + | | 24278g0011 | | | + | | 005012m.g | | + | | AT5G26250 | | - | | 1016148001 | | + | | 11g00710 | | + | | 07g10590 | | + | |  |  |
| PGSC0003DMG400014215 | + | Solyc03g117730.2 | | | - | DF362996.1 | | | + | 03g000561 | + | | 26409g0013 | | | + | | 007034m.g | | + | | AT1G16070 | | + | | 1016095001 | | + | | 13g44890 | | + | | 02g08310 | | + | |  |  |
| PGSC0003DMG400021152 | + | Solyc02g069250.2 | | | - | - | | | ? | 02g001298 | + | | 19196g0005 | | | + | | 008846m.g | | + | | AT4G39330 | | + | | 1009972001 | | - | | 14g40170 | | + | | 04g15920 | | + | |  |  |
| PGSC0003DMG400022258 | - | Solyc07g065980.2 | | | + | DF358743.1 | | | + | 07g002427 | + | | 01592g0014 | | | + | | 001855m.g | | - | | AT1G55740 | | - | | 1014778001 | | + | | 14g01430 | | - | | 08g38710 | | + | |  |  |
| PGSC0003DMG400022442 | + | Solyc02g078430.2 | | | - | - | | | ? | - | ? | | 07304g0006 | | | + | | 015869m.g | | + | | AT4G21720 | | + | | 1002932001 | | + | | 13g24910 | | + | | 04g58380 | | + | |  |  |
| PGSC0003DMG400023424 | - | Solyc05g054580.2 | | | + | DF357359.1 | | | + | 06g000359 | + | | 19795g0001 | | | - | | - | | ? | | AT3G11250 | | + | | 1032857001 | | + | | 14g06630 | | - | | 12g03880 | | + | |  |  |
| PGSC0003DMG400025257 | + | Solyc01g086750.2 | | | - | DF364137.1 | | | + | 00g000390 | + | | 42740g0010 | | | + | | 000207m.g | | + | | AT3G52140 | | ? | | 1032046001 | | + | | 08g16180 | | + | | 02g48620 | | + | |  |  |
| **Imprecise indel** |  |  | | |  |  | | |  |  |  | |  | | |  | |  | |  | |  | |  | |  | |  | |  | |  | |  | |  | |  |  |
| PGSC0003DMG400000276 | - | Solyc12g008940.1 | | | + | DF357624.1 | | | + | 09g000183 | ? | | 08501g0010 | | | ? | | 008627m.g | | + | | AT2G19480 | | - | | 1016870001 | | + | | 17g06170 | | + | | - | | ? | |  |  |
| PGSC0003DMG400005774 | | | + | Solyc08g007920.1 | | | - | - | | | | ? | | 01g000311 | + | | 00193g0022 | | + | | 008113m.g | | + | | - | | ? | | - | | ? | | 01g42620 | | + | | - | | ? |
| PGSC0003DMG400006006 | | | + | Solyc04g007270.2 | | | - | DF365660.1 | | | | + | | - | ? | | 15128g0005 | | ? | | - | | ? | | AT1G33420 | | ? | | 1000247001 | | ? | | 10g31980 | | ? | | 01g65600 | | ? |
| PGSC0003DMG400006365 | | | + | Solyc04g071660.2 | | | - | DF364761.1 | | | | + | | 04g001269 | + | | 14171g0012 | | + | | - | | ? | | - | | ? | | - | | ? | | - | | ? | | - | | ? |
| PGSC0003DMG400016267 | | | + | Solyc06g054030.2 | | | - | - | | | | ? | | 00g002255 | + | | 18718g0011 | | + | | 023041m.g | | + | | AT1G05450 | | + | | 1018010001 | | - | | 10g39190 | | + | | 03g57980 | | + |
| PGSC0003DMG400027761 | | | + | Solyc05g025890.1 | | | - | - | | | | ? | | - | ? | | 21003g0115 | | + | | - | | ? | | - | | ? | | 1015086001 | | + | | - | | ? | | - | | ? |
| PGSC0003DMG400018521 | | | - | Solyc03g082870.2 | | | + | DF357445.1 | | | | + | | 03g001972 | ? | | 59269g0007 | | ? | | 027154m.g | | ? | | AT5G24320 | | ? | | 1038588001 | | ? | | 06g13660 | | ? | | 03g02440 | | ? |
| PGSC0003DMG400026971 | | | + | Solyc06g072100.1 | | | - | DF357319.1 | | | | + | | - | ? | | 29362g0001 | | + | | 002042m.g | | + | | AT1G17840 | | + | | 1024228001 | | + | | 16g33470 | | + | | 10g35180 | | + |
| PGSC0003DMG400035888 | | | - | Solyc07g009110.1 | | | + | - | | | | ? | | 09g001914 | ? | | 26384g0008 | | - | | 008358m.g | | + | | - | | ? | | 1010300001 | | ? | | - | | ? | | - | | ? |
| PGSC0003DMG401027564 | | | + | Solyc04g072400.2 | | | - | BAUE01053985.1 | | | | + | | 04g001175 | ? | | 38989g0005 | | ? | | 008619m.g | | ? | | AT1G36320 | | - | | 1008964001 | | ? | | 09g02440 | | ? | | 01g20110 | | ? |
| PGSC0003DMG400003903 | | | + | Solyc09g008530.1 | | | - | DF357317.1 | | | | ? | | 09g002281 | ? | | 18542g0006 | | + | | 001004m.g | | + | | - | | ? | | 1033709001 | | ? | | 13g21083 | | + | | 03g01710 | | ? |
| PGSC0003DMG400005572 | | | - | Solyc01g110380.2 | | | + | DF357388.1 | | | | ? | | - | ? | | 52706g0005 | | - | | 001175m.g | | + | | AT2G21300 | | + | | 1024172001 | | + | | 12g04120 | | + | | 04g45580 | | + |
| PGSC0003DMG400010619 | | | + | Solyc04g077790.2 | | | - | DF357290.1 | | | | ? | | - | ? | | 27488g0004 | | + | | 010806m.g | | + | | AT5G22370 | | + | | 1009339001 | | ? | | 04g04870 | | + | | 02g34950 | | + |
| PGSC0003DMG400012167 | | | - | Solyc08g081890.2 | | | + | DF357222.1 | | | | ? | | - | ? | | 16857g0026 | | - | | 000196m.g | | + | | AT3G59140 | | + | | 1028722001 | | + | | 10g37160 | | + | | 06g06440 | | + |
| PGSC0003DMG400012403 | | | - | Solyc07g062840.2 | | | + | - | | | | ? | | 07g002159 | + | | 14197g0005 | | - | | 009356m.g | | + | | AT5G53950 | | + | | 1014287001 | | + | | 13g34950 | | + | | 06g23650 | | - |
| PGSC0003DMG400015280 | | | - | Solyc03g019930.2 | | | + | - | | | | ? | | 03g001431 | + | | 09480g0024 | | - | | 006601m.g | | ? | | AT1G73380 | | ? | | 1024248001 | | + | | - | | ? | | 10g34810 | | ? |
| PGSC0003DMG400018336 | | | + | Solyc07g053900.2 | | | - | DF359643.1 | | | | ? | | 07g001743 | + | | 33220g0010 | | + | | 010863m.g | | + | | AT2G20670 | | + | | 1005210001 | | + | | 12g12870 | | - | | 01g54340 | | + |
| PGSC0003DMG400034307 | | | - | Solyc05g015610.2 | | | + | DF361132.1 | | | | ? | | 11g001031 | + | | 11469g0014 | | - | | 000769m.g | | + | | AT1G27320 | | + | | 1010502001 | | + | | 08g11060 | | + | | 01g69920 | | + |
| PGSC0003DMG401012908 | | | + | Solyc04g076120.2 | | | - | DF357757.1 | | | | ? | | - | ? | | 36239g0006 | | + | | 005845m.g | | + | | - | | ? | | 1009319001 | | + | | 15g09700 | | + | | 10g01110 | | + |

“+” indicates the presence of the intron, “-” indicates the absence of the intron, and “?” indicates that no confident result was observed.

The space limitations in this table required us to shorten the transcript names by omitting a few characters at the beginning of the names of the following species: *C. annuum* (Capana), *N. benthamiana* (NbS000), *M. guttatus* (mgv1a), *V. vinifera* (GSVIVG0), *G. max* (Glyma), and *O. sativa* (LOC_Os). For example, 11g00710 was abbreviated from *Glyma11g00710*.

Data S1. Aligned sequences showing intron variations in *S. tuberosum* and *S. lycopersicum.*

**Contents**

[Instructions 7](#_Toc407350386)

[Precise Intron loss 7](#_Toc407350387)

[*Solyc02g087120.2* 7](#_Toc407350388)

[*PGSC0003DMG400004708* 8](#_Toc407350389)

[*PGSC0003DMG400009895* 9](#_Toc407350390)

[*PGSC0003DMG400010092* 10](#_Toc407350391)

[*PGSC0003DMG400013384* 11](#_Toc407350392)

[*Solyc03g117730.2* 12](#_Toc407350393)

[*Solyc02g069250.2* 13](#_Toc407350394)

[*PGSC0003DMG400022258* 14](#_Toc407350395)

[*Solyc02g078430.2* 15](#_Toc407350396)

[*PGSC0003DMG400023424* 16](#_Toc407350397)

[*Solyc01g086750.2* 17](#_Toc407350398)

[Imprecise Intron loss 18](#_Toc407350401)

[*PGSC0003DMG400000276* 18](#_Toc407350402)

[*Solyc08g007920.1* 19](#_Toc407350403)

[*Solyc04g007270.2* 20](#_Toc407350404)

[*Solyc05g025890.1* 21](#_Toc407350405)

[*Solyc06g054030.2* 22](#_Toc407350406)

[*Solyc04g071660.2* 23](#_Toc407350407)

[Exon deletion 25](#_Toc407350415)

[*Solyc09g016940.2* 25](#_Toc407350416)

[De-exonizaion 25](#_Toc407350411)

[*PGSC0003DMG400004043* 26](#_Toc407350412)

[Intronizaion 28](#_Toc407350408)

[*PGSC0003DMG400022716* 28](#_Toc407350409)

[*Solyc06g068980.2* 29](#_Toc407350410)

[Exonization 30](#_Toc407350413)

[*Solyc10g005290.2* 30](#_Toc407350414)

# Instructions

Intronic sequences are presented in lowercase and exonic sequences are in uppercase.

Indels that occurred before the divergence of *Solanum lycopersicum* and *Solanum tuberosum* and those that occurred in outgroup species (e.g., *Capsicum annuum*) were not relevant to our study and would lead to confusion if their alignments were presented. Therefore, these indels are not shown in the following alignments. The alignments containing unabridged sequences are available upon request ([dengkeniu@hotmail.com](mailto:dengkeniu@hotmail.com), [dkniu@bnu.edu.cn](mailto:dkniu@bnu.edu.cn)).

# Precise Intron loss

## *Solyc02g087120.2*

*S. lycopersicum* AGCATTTGAAGCTGCTTTCAACTACATAAACCAATGCGTGGAATGCTCAG----------------------------------------

*S. tuberosum*  AGCATTTCAAGCTGCTTTCAACTACATAAACAAATGCGTGGAATGCTCAGgtatatatagactctgttttaatcttgatgggttcaattt

*C. annuum*  AGCATTTCAAGCTGCTTTCAACCACATTAACGAATGTGTGGAATGCTCAGgtatagacggattcagttttaagtgtgactccttcaacct

*S. lycopersicum* -------------------------------------------------GTATTGTTGTGCTTTTGAGGACATTCTCCGCCGGTCAAT

*S. tuberosum*  ttaaaattatcataaatacgttgaactcattcctaaaaagtctgaatagGTATTGTTGTGCTTTTGAGGACATTCTCCGCTGGTCAAT

*C. annuum*  ttgaaattatgattgatacattcaattcattactaaaagttttgactagGTATTTTTGTACTTTTGAGGACATTATCGGCAAGGCAAT

The *S. lycopersicum* gene *Solyc02g087120.2* has lost an intron. The active expression of the gene is supported by SRR567999.sra.26776963, SRR567999.sra.18975203, SRR567999.sra.36971767, SRR567999.sra.34940275, SRR567999.sra.28229774, SRR567999.sra.2594523, SRR567999.sra.10408794, SRR567999.sra.41783973, SRR567999.sra.23562160, and SRR567999.sra.2191170 (> 10 RNA-Seq reads, others are not shown).

The successful splicing of the target intron in *S. tuberosum* is supported by SRR866259.sra.12819862, SRR866259.sra.17074846, SRR866259.sra.9195133, SRR866259.sra.4413335, SRR866275.sra.10300237, SRR866275.sra.1925548, SRR184103.sra.1104076, SRR184103.sra.1508965, SRR184103.sra.3189373, and SRR184103.sra.5984291 (> 10 RNA-Seq reads, others are not shown).

The successful splicing of the target intron in *C. annuum* is supported by SRR771938.26255336, SRR771940.27815396, SRR771940.15487546, SRR771949.18067136, SRR771949.4107671, SRR771949.6231545, SRR771950.13272832, SRR771950.16542082, SRR771950.23023481, SRR771950.25105555, SRR771950.7175832, and SRR771950.989250 (> 10 RNA-Seq reads, others are not shown).

The assembly of the variation site in *S. lycopersicum* was supported by WGS reads. First, we found eight WGS reads of *S. lycopersicum* crossing the variation site in *S. lycopersicum*: ERR327647.15659876, ERR327647.32058204, ERR327646.5548945, ERR327646.10223513, ERR327646.18091452, ERR327647.29909692, ERR327646.5261063, and ERR327647.28423864. In addition, we found > 10 *S. lycopersicum* WGS reads that matched the regions flanking the target variation site (1 kb at each side) in *S. tuberosum* (ERR327646.18852243, ERR327646.30009875, ERR327646.18307459, ERR327646.19177146, ERR327646.18091452, ERR327646.15067102, ERR327648.29512903, ERR327648.8838204, ERR327648.31681795, and ERR327650.17214557, others are not shown) but no WGS reads of *S. lycopersicum* that matched the target intron of *S. tuberosum*, which indicates that the intron is absent in *S. lycopersicum*.

## *PGSC0003DMG400004708*

*S. tuberosum*  CTCTCCTAATGCAGGATCATGTGAACATACAATCAATACATTGAGATATGCTGACAG---------------------------------

*S. lycopersicum* CTCTCCTAATGCAGGATCATGTGAACATACAATTAATACATTGAGATATGCTGACAGgtaaattttttctttaagcgtgggttgatttta

*C. annuum*  CTCTCCTAATGCAGGATCATGCGAACATACACTCAATACATTGAGATATGCTGACAGgtaaaca--------------gggttgatttta

*S. tuberosum*  --------------------------------------------GGTGAAAAGTCTATCCAAAAGTGGAAACACAAAGAAAGATCA

*S. lycopersicum* ttgtttcctatcgagcatgggttaagctaaaatgtgataaacagGGTGAAAAGTCTATCGAAAAGTGGAAACACAAAGAAAGATCA

*C. annuum*  ttgtttcctattaaacatgggttatactaacatgtgattaacagGGTGAAAAGTCTATCCAAAAGTGGAAACACAAAGAAAGATCA

The *S. tuberosum* gene *PGSC0003DMG400004708* has lost an intron. The active expression of the gene is supported by SRR184103.sra.5862237, SRR184103.sra.2689134, SRR184103.sra.1179613, SRR184103.sra.2637850, SRR184103.sra.4091162, SRR184103.sra.1091682, SRR184103.sra.2888397, SRR184103.sra.4552489, SRR184103.sra.6380434, and SRR184103.sra.5678875 (> 10 RNA-Seq reads, others are not shown).

The successful splicing of the target intron in *S. lycopersicum* is supported by SRR768862.sra.16199980, SRR768862.sra.11207021, SRR768862.sra.17652128, SRR768864.sra.9487759, SRR768864.sra.13908729, SRR568000.sra.25085132, SRR568000.sra.6892730, SRR568000.sra.25870344, SRR568000.sra.42383038, SRR568000.sra.5804134 (> 10 RNA-Seq reads, others are not shown) and the EST asmbl_2305.tomatov23pasa_pasa4.

The successful splicing of the target intron in *C. annuum* is supported by SRR771940.9677107, SRR771940.19251806, SRR771940.19469970, SRR771940.5530358, SRR771940.12826644, SRR771940.23813825, SRR771940.13659677, SRR771940.25043171, SRR771940.21526012, and SRR771940.17646737 (> 10 RNA-Seq reads, others are not shown).

The assembly of the variation site in *S. tuberosum* was supported by WGS reads. First, we found nine WGS reads of *S. tuberosum* crossing the variation site in *S. tuberosum*: SRR307648.20474227, SRR307648.3338948, SRR307648.20145858, SRR307648.5033377, SRR307648.3569982, SRR307648.8921627, SRR307626.9139266, SRR307626.183486, and SRR307626.9739149. In addition, we found > 10 *S. tuberosum* WGS reads that matched the regions flanking the target variation site (1 kb at each side) in *S. lycopersicum* (SRR307587.1093413, SRR307587.3029773, SRR307587.3186372, SRR307587.3310308, SRR307587.3338431, SRR307587.637670, SRR307598.1042899, SRR307598.1234905, SRR307598.1748077, and SRR307598.1748077, others are not shown) but no WGS reads of *S. tuberosum* that matched the target intron of *S. lycopersicum*, which indicates that the intron is absent in *S. tuberosum*.

## *PGSC0003DMG400009895*

*S. tuberosum*  GCTACCAGAATTATCGGCGTTGGTAGGAACTCTGACAAGTTTGAAATAG-----------------------------------------

*S. lycopersicum* GCTACCAGGATTATCGGTGTTGATATAAACTCTAACAAGTTTGAAATAGgtaagtgctctgtagagtcaattaacagtactactttttgt

*C. annuum*  GCTACCAGAATTATTGGCGTTGATATAAACTCTGACAAGTTTGAAATAGgtaagcatcttgagaaattgattaacagtaatacttctcct

*S. tuberosum*  ------------------------------GGAAGCAGTTTGGAGTTACTGAGTTTGTCAATTCC

*S. lycopersicum* tgttgcttttagctttctcattttatacagGGAAGCAATTTGGAATTACTGAATTTGTCAATTCC

*C. annuum*  ttttgtttttacctttctcatttgacacagGGAAGCAGTTTGGAATTACTGAATTTGTCAATTCC

The *S. tuberosum* gene *PGSC0003DMG400009895* has lost an intron. The active expression of the gene is supported by SRR184103.sra.2574239, SRR184103.sra.4078619, SRR184103.sra.676812, SRR184103.sra.1758294, SRR184103.sra.2042890, SRR184103.sra.2048358, SRR184103.sra.3320684, SRR184103.sra.3733944, SRR184103.sra.5209839, and SRR184103.sra.3608863 (> 10 RNA-Seq reads, others are not shown).

The successful splicing of the target intron in *S. lycopersicum* is supported by SRR567999.sra.6262664.

The successful splicing of the target intron in *C. annuum* is supported by SRR771940.11425188, SRR771940.25966931, SRR771940.1479626, SRR771940.10209573, SRR771940.76147, SRR771929.17744363, SRR771929.40444337, SRR771929.17982231, SRR771929.20434319, and SRR771929.952110 (> 10 RNA-Seq reads, others are not shown).

The assembly of the variation site in *S. tuberosum* was supported by WGS reads. First, we found eight WGS reads of *S. tuberosum* crossing the variation site in *S. tuberosum*: SRR307648.15512755, SRR307648.5444053, SRR307648.6552849, SRR307648.14438000, SRR307648.19262306, SRR307626.7485211, SRR307626.7590277, and SRR307611.7644708. In addition, we found > 10 *S. tuberosum* WGS reads that matched the regions flanking the target variation site (1 kb at each side) in *S. lycopersicum* (SRR307587.140122, SRR307587.304049, SRR307587.3396069, SRR307587.3396069, SRR307587.678160, SRR307605.349201, SRR307605.5290736, SRR307605.7999321, SRR307605.8878926, and SRR307605.8878926, others are not shown) but no WGS reads of *S. tuberosum* that matched the target intron of *S. lycopersicum*, which indicates that the intron is absent in *S. tuberosum*.

## *PGSC0003DMG400010092*

*S. tuberosum*  CAAGAAGCTTTTGGCATATGGTTACCTTGTTCGAGTTATTATTCAAAATCAAG-------------------------------------

*S. lycopersicum* CAAGAAGCTTTTGGCATATGGTTACCTTGTTCGAGTTATTATTCAAAATCAAGgtacacaagccagcaaccctctttccttatacttgtg

*C. annuum*  CATGAAGCTTTTGGCATGTGGTTACCTTGTTCGAGTTATTATTGAAAACCAAGgt--actagctagcaaccctttttccttatacttgtg

*S. tuberosum*  -----------------------------------------------------CTAATCTTGAGGACATGAAGGAGCTGATGAGGGAAGA

*S. lycopersicum* cagttccaatttgtgtcttgctaaaacttacaagttactcctgttttaagtagCTAATCTTGAGGATATGAAGGAGCTAATGAGGGAAGA

*C. annuum*  tagattcaatttttgtcttgctaaaagttacaagttactcctg-tatgagcagCTAATCTTGAGGACATGGAGGAACTGATTAGGGAAGA

The *S. tuberosum* gene *PGSC0003DMG400010092* has lost an intron. The active expression of the gene is supported by SRR184103.sra.2993070, SRR184103.sra.3968551, SRR184103.sra.6219650, SRR184103.sra.4361777, SRR184103.sra.5471952, SRR184103.sra.2424132, SRR184103.sra.5514764, SRR184103.sra.4836878, SRR184103.sra.5042447, and SRR184103.sra.4485828 (> 10 RNA-Seq reads, others are not shown).

The successful splicing of the target intron in *S. lycopersicum* is supported by SRR768860.sra.18720106, SRR768860.sra.93552, SRR768860.sra.7805669, SRR768860.sra.14304796, SRR768860.sra.6203692, SRR768835.sra.18453931, SRR768835.sra.9423871, SRR768835.sra.7004864, SRR768835.sra.14879339, SRR768835.sra.15876796 (> 10 RNA-Seq reads, others are not shown) and EST asmbl_3299.tomatov23pasa_pasa5.

The successful splicing of the target intron in *C. annuum* is supported by SRR771929.1768441, SRR771929.18587168, SRR771929.28629004, SRR771929.28838191, SRR771929.29476067, SRR771941.16596927, SRR771941.9753189, SRR771953.1872698, SRR771953.28896483, and SRR771925.17344187 (> 10 RNA-Seq reads, others are not shown).

The assembly of the variation site in *S. tuberosum* was supported by WGS reads. First, we found ten WGS reads of *S. tuberosum* crossing the variation site in *S. tuberosum*: SRR307648.17629287, SRR307648.1734989, SRR307648.15307116, SRR307648.4436690, SRR307648.438769, SRR307648.3848581, SRR307648.3671608, SRR307626.4350340, SRR307611.5066637, and SRR307611.4022060. In addition, we found > 10 *S. tuberosum* WGS reads that matched the regions flanking the target variation site (1 kb at each side) in *S. lycopersicum* (SRR307587.1360298, SRR307587.2795247, SRR307587.2813445, SRR307587.3560174, SRR307587.3736611, SRR307598.3930177, SRR307598.3959242, SRR307598.4004596, SRR307598.4271637, and SRR307598.4829249, others are not shown) but no WGS reads of *S. tuberosum* that matched the target intron of *S. lycopersicum*, which indicates that the intron is absent in *S. tuberosum*.

## *PGSC0003DMG400013384*

*S. tuberosum*  ATTGGAAGGAGAAAGTTGCTTCTCCAAGCTTGTTGCCAAATGTTAATTTCTCAG------------------------------------

*S. lycopersicum* GTTGGAAGGAGAAAATTGCTTCTCCAAGCTTGTTGTCAAATGTTAATTTCTCAGgtatgatatcatatactatttcctgtctacatgatt

*C. annuum*  GTTGGAAGGAAAAAATTGCTCCTCCAAGCTTGTTGCCAGATGTTGATCTCTCATgtacgaaatctcttactaacgcttgcctaggtgagc

*S. tuberosum*  -------------------------------------------TTGGCAATAGGAGCTATTTTGGTAACAAATTTGGAGGAGACAGGAAC

*S. lycopersicum* attcatcaaaactgagaatttatatttggtttgaatggtgcagTTGGCAATTGGAGCTATTTTGGTAACAAATTTGGAGGAGACAGGAAC

*C. annuum*  atccatcttgattagttttctgtattttttttgaattttgcagCTGGCAATTGGAATAATATTGACAACCAGTTTGAGAGAGACAGGATC

The *S. tuberosum* gene *PGSC0003DMG400013384* has lost an intron. The active expression of the gene is supported by SRR184103.sra.1421763, SRR184103.sra.6110747, SRR184103.sra.6110747, and SRR184104.sra.5871962.

The successful splicing of the target intron in *S. lycopersicum* is supported by SRR786507.sra.4647203, SRR567999.sra.24034672, and SRR567999.sra.16379114.

The successful splicing of the target intron in *C. annuum* is supported by RR771929.12345032, SRR771929.15035418, SRR771929.19755342, SRR771929.15504735, SRR771929.33896986, SRR771924.4517326, SRR771949.17758612, SRR771949.875865, SRR771949.24600548, and SRR771949.8928647 (> 10 RNA-Seq reads, others are not shown).

The assembly of the variation site in *S. tuberosum* was supported by WGS reads. First, we found five WGS reads of *S. tuberosum* crossing the variation site in *S. tuberosum*: SRR307648.16590001, SRR307648.15678166, SRR307648.1554184, SRR307626.9824560, and SRR307626.9452583. In addition, we found > 10 *S. tuberosum* WGS reads that matched the regions flanking the target variation site (1 kb at each side) in *S. lycopersicum* (SRR307587.1780932, SRR307587.1790567, SRR307587.1880018, SRR307587.3164601, SRR307587.5614181, SRR307598.2254530, SRR307598.2475541, SRR307598.2519883, SRR307598.2585494, and SRR307598.3701017, others are not shown) but no WGS reads of *S. tuberosum* that matched the target intron of *S. lycopersicum*, which indicates that the intron is absent in *S. tuberosum*.

## *Solyc03g117730.2*

*S. lycopersicum* GGACAAAGTGAATCAGTTGTTTTCAAAGATTCCTCATTACAATAAG--------------------------------------------

*S. tuberosum*  GGACAAAGTGAATCAGTTGTTTTCGAAGATTCCTCATTACAATAAGgtataaatatctgttttgtataaccgtttgaaaatgctttgagt

*C. annuum*  GGACAAAGTGAATCAGTTGTTTTCAAAGATTCCTCATTACAATAAGgtataaatatttgtcttgaatggctgtttcgaagttttttgagt

*S. lycopersicum* ---------------------------------------------------GTCTCAAGACAGTATGAGTTAGATTTTAGAGATA

*S. tuberosum*  ttgtttttgaagattcctcacatctaatatgcaattatttggtacatgtagGTCTCAAGACAGTATGAGTTAGATTTTAGAGATA

*C. annuum*  cgagtcctcattttttagcacatagcatatgcaattatttggtacatgtagGTCTCAAGACAGTATGAGTTAGATTTTAGAGATA

The *S. lycopersicum* gene *Solyc03g117730.2* has lost an intron. The active expression of the gene is supported by SRR567999.sra.40819924, SRR567999.sra.15269443, SRR567999.sra.18368446, SRR567999.sra.43606716, SRR567999.sra.19907406, SRR567999.sra.19302489, SRR567999.sra.30008375, SRR567999.sra.36990448, SRR567999.sra.43254514, and SRR567999.sra.5392937 (> 10 RNA-Seq reads, others are not shown).

The successful splicing of the target intron in *S. tuberosum* is supported by SRR866275.sra.2930349, SRR184103.sra.3566514, SRR184103.sra.3956410, SRR184104.sra.2086501, SRR184104.sra.5501114, and SRR184104.sra.128498.

The assembly of the variation site in *S. lycopersicum* was supported by WGS reads. First, we found seven WGS reads of *S. lycopersicum* crossing the variation site in *S. lycopersicum*: ERR327647.10483641, ERR327647.31016313, ERR327647.6648692, ERR327647.13113674, ERR327647.23739195, ERR327646.10976272, and ERR327646.12358043. In addition, we found > 10 *S. lycopersicum* WGS reads that matched the regions flanking the target variation site (1 kb at each side) in *S. tuberosum* (ERR327646.887758, ERR327646.19940385, ERR327646.14472422, ERR327646.19173754, ERR327646.10164903, ERR327647.2221132, ERR327647.14888273, ERR327647.13626699, ERR327648.31426393, and ERR327648.1915223, others are not shown) but no WGS reads of *S. lycopersicum* that matched the target intron of *S. tuberosum*, which indicates that the intron is absent in *S. lycopersicum*.

## *Solyc02g069250.2*

*S. lycopersicum* AAGCCCATGGACCTTCCATCATTTCCATTAATATTTG-----------------------------------------------------

*S. tuberosum*  AAGCCCATGGACCTTCCATCATTTCCATTAATATTTGgtaagcaatattaacaacactttattttatatttttaaggattgtatttgaag

*C. annuum*  AAGCCCATGGACCTTCCATCATTTCCATTAATATTTGgtaagtaatattaagaacacttcattctat-catttaaggattgtgttt-aag

*S. lycopersicum* ---------------------------------------GTAAAAGAACAGTGAAAGGGAGCATGATT

*S. tuberosum*  atttctttaataaataaccctttaaattgtgaaatgcagGTAAGAGAACTGTGAAAGGAAGCATGATT

*C. annuum*  attttcttgataataaccccttaaaattgtgaaatgcagGTAAGAGAACTGTGAAAGGAAGCATGATT

The *S. lycopersicum* gene *Solyc02g069250.2* has lost an intron. The active expression of the gene is supported by SRR567999.sra.19410921, SRR567999.sra.19410921, SRR567999.sra.20810198, SRR567999.sra.20810198, SRR567999.sra.15797938, SRR567999.sra.41494214, SRR567999.sra.19668025, SRR567999.sra.35623996, SRR567999.sra.18258451, and SRR567999.sra.16121420 (> 10 RNA-Seq reads, others are not shown).

The successful splicing of the target intron in *S. tuberosum* is supported by SRR184104.sra.4805972, SRR866259.sra.8023079, SRR866259.sra.7870388, SRR866259.sra.6379908, SRR866266.sra.15530129, SRR866268.sra.12258185, SRR866268.sra.12958796, SRR866268.sra.10263983, SRR866268.sra.1021404, and SRR866275.sra.3826256 (> 10 RNA-Seq reads, others are not shown).

The successful splicing of the target intron in *C. annuum* is supported by SRR771940.9804039, SRR771940.6399589, SRR771940.15973889, SRR771940.16797213, SRR771940.1083491, SRR771929.40761818, SRR771949.10800832, SRR771949.6595915, SRR771949.23310003, and SRR771949.21857951 (> 10 RNA-Seq reads, others are not shown).

The assembly of the variation site in *S. lycopersicum* was supported by WGS reads. First, we found eight WGS reads of *S. lycopersicum* crossing the variation site in *S. lycopersicum*: ERR327647.2385273, ERR327647.32054867, ERR327647.13004399, ERR327646.24029806, ERR327646.3351924, ERR327646.3336830, ERR327647.2623824, and ERR327647.8083652. In addition, we found > 10 *S. lycopersicum* WGS reads that matched the regions flanking the target variation site (1 kb at each side) in *S. tuberosum* (ERR327646.19267573, ERR327646.4602443, ERR327646.16906199, ERR327646.11872707, ERR327646.7514108, ERR327651.27014579, ERR327653.22889401, ERR327654.28240856, ERR327654.1910544, and ERR327655.16939997, others are not shown) but no WGS reads of *S. lycopersicum* that matched the target intron of *S. tuberosum*, which indicates that the intron is absent in *S. lycopersicum*.

## *PGSC0003DMG400022258*

*S. tuberosum*  ATGGGACTTCGACATGTTGTTACCAATATCAAAGACCAACACAATTTGAA----------------------------------------

*S. lycopersicum* ATGGGACTTCGACATGTTGTTACCAATATCAAAGACCAACACAATTTGAAgtatgttcttcaatatcttcaactatcaggtgctgtcctt

*C. annuum*  ATGGGACTTCGACATGTTGTTGCCAATATCAAAGACCAACACGATTTGAAgtatgttctttaatatcgtcaactattaggtgctatcctt

*S. tuberosum*  ----------------------------------------------GTATGTGTACATGTGGCATGCACTTGCTGGTTACTGGGGTGGTG

*S. lycopersicum* ttaattatcaactaagcgttttccttctttggatcatgcactttagGTATGTGTACATGTGGCATGCACTTGCTGGTTACTGGGGCGGTG

*C. annuum*  ttaattatccactaatccatttacttctttaaaccgtgaactttagGTACGTGTATGTGTGGCATGCACTCGCTGGTTACTGGGGCGGTA

The *S. tuberosum* gene *PGSC0003DMG400022258* has lost an intron. The active expression of the gene is supported by SRR184103.sra.337335, SRR184103.sra.6461400, SRR184103.sra.3447800, SRR184103.sra.3009173, SRR184103.sra.87565, SRR184103.sra.372861, SRR184103.sra.3329799, SRR184103.sra.4213157, SRR184103.sra.146925, and SRR184103.sra.171806 (> 10 RNA-Seq reads, others are not shown).

The successful splicing of the target intron in *S. lycopersicum* is supported by SRR567999.sra.16664427, SRR567999.sra.2822305, SRR567999.sra.17624013, SRR567999.sra.35865180, SRR567999.sra.21614563, SRR768835.sra.16173508, SRR768835.sra.10111955, SRR768835.sra.4713960, SRR768835.sra.13378203, SRR768835.sra.8869088 (> 10 RNA-Seq reads, others are not shown) and EST asmbl_2728.tomatov23pasa_pasa10.

The successful splicing of the target intron in *C. annuum* is supported by SRR771940.6997337, SRR771940.433544, SRR771940.13526062, SRR771940.25703227, SRR771940.17871035, SRR771940.3401133, SRR771940.2057897, SRR771940.17398784, SRR771940.28200784, and SRR771940.10916040 (> 10 RNA-Seq reads, others are not shown).

The assembly of the variation site in *S. tuberosum* was supported by WGS reads. First, we found six WGS reads of *S. tuberosum* crossing the variation site in *S. tuberosum*: SRR307648.19282777, SRR307648.8239571, SRR307648.4978211, SRR307626.1544704, SRR307626.8918501, and SRR307626.9512124. In addition, we found > 10 *S. tuberosum* WGS reads that matched the regions flanking the target variation site (1 kb at each side) in *S. lycopersicum* (SRR307587.261273, SRR307587.274338, SRR307587.3743600, SRR307587.3743600, SRR307587.403133, SRR307598.2756432, SRR307598.2810639, SRR307598.3436021, SRR307598.417568, SRR307598.4375533, others are not shown) but no WGS reads of *S. tuberosum* that matched the target intron of *S. lycopersicum*, which indicates that the intron is absent in *S. tuberosum*.

## *Solyc02g078430.2*

*S. lycopersicum* ATGGAGTGCTTGTATTGATCGCGCCCCTGGTTCTGCTACCCACTCTGAG-----------------------------------------

*S. tuberosum*  ATGGAGTGCTTGTATTGATCGCGCCCCTGGTTCTGCTAGCCACTCTGAGgtttaccatctcttcatttcattttattaccctgaaatttg

*A. thaliana*  ATGGACTGCTGTTATAGATCGTTCTCCTGGAGATGCCAAATACTCTGAGgtaattcaccaatgaatctttttcttcaaaaaagagaaatg

*S. lycopersicum* --------------------------------------------------GATTGTCATCGCGCTTGTGTAGCTGGCTGTGGTTTCAAG

*S. tuberosum*  gtgtcttttaacacttgttattcaacatgtgtgttcttttatggatgcagGATTGTCATCGCGCTTGTGTAGCTGGTTGTGGTTTCAAG

*A. thaliana*  gactgaaccgtatctaattgttgtacatgtaactctgtttttaaattcagGAATGCTTTCACGCATGTGTAGCAGGCTGTGGTTACAAG

The *S. lycopersicum* gene *Solyc02g078430.2* has lost an intron. The active expression of the gene is supported by SRR568000.sra.22009363, SRR568000.sra.10546282, SRR568000.sra.10546282, SRR567999.sra.4484876, and SRR567999.sra.4484876.

The successful splicing of the target intron in *A. thaliana* is supported by SRR394082.sra.47640565, SRR394082.sra.20572642, SRR394082.sra.12883965, SRR394082.sra.48473847, SRR394082.sra.27270233, SRR394082.sra.12956996, SRR394082.sra.21875986, SRR394082.sra.11343651, SRR394082.sra.20484042, SRR394082.sra.13256263 (> 10 RNA-Seq reads, others are not shown) and ESTs asmbl_3524.p5arabi3 and asmbl_3525.p5arabi3.

The assembly of the variation site in *S. lycopersicum* was supported by WGS reads. First, we found six WGS reads of *S. lycopersicum* crossing the variation site in *S. lycopersicum*: ERR327647.3744582, ERR327647.16032360, ERR327646.25497061, ERR327646.11320293, ERR327647.30055630, and ERR327646.32800498. In addition, we found > 10 *S. lycopersicum* WGS reads that matched the regions flanking the target variation site (1 kb at each side) in *S. tuberosum* (ERR327646.21277633, ERR327646.18909797, ERR327646.25420918, ERR327646.5552236, ERR327646.27510268, ERR327648.18760323, ERR327648.30859252, ERR327648.33238066, ERR327649.12587970, and ERR327649.30643887, others are not shown) but no WGS reads of *S. lycopersicum* that matched the target intron of *S. tuberosum*, which indicates that the intron is absent in *S. lycopersicum*.

## *PGSC0003DMG400023424*

*S. tuberosum*  CCGACTATTCTTTCCCTCTAGCTGACAAAGTGAAGGAATACCTCGCC-------------------------------------------

*S. lycopersicum* CCGACTACTCTTTCCCTCTCGCTGACAAAGTGAAGGAATACCTCGCGgtatgttcttaatcccaatgttccaaagatgtaatatataaac

*S. melongena*  CCGACTACTCTTTCCCTCTGGCTGACAAAGTGAAGGAGTACCTCGCGgtatgttttcaatgacaatggctcaaagctatcttatattaaa

*S. tuberosum*  ---------------------------------------GATCCTAGCAAGTTT---GCTGTTGCTGCTGCTCCTGCTGCAGCTGCTGGT

*S. lycopersicum* agttagttaactttgtctcaactattcttgcacttgcagGATCCAAGTAAGTTTGCCGCTGTTGCTGCCGCTCCTGCTGCAGCTGCTGGT

*S. melongena*  tgttagtt-acattctcttaactacttttgcacctgcagGATCCTAGCAAGTTT---GCTGTTGCTGCTGCTCCGGTTGCAGCTGCTGGT

The *S. tuberosum* gene *PGSC0003DMG400023424* has lost an intron. The active expression of the gene is supported by SRR184103.sra.4122424, SRR184103.sra.4313926, SRR184103.sra.5121688, SRR184103.sra.164925, SRR184103.sra.282833, SRR184103.sra.443929, SRR184103.sra.4421513, SRR184103.sra.6508918, SRR184103.sra.208741, and SRR184103.sra.3495601 (> 10 RNA-Seq reads, others are not shown).

The successful splicing of the target intron in *S. lycopersicum* is supported by SRR568000.sra.14701604, SRR568000.sra.28990359, SRR568000.sra.3068027, SRR568000.sra.4288797, SRR568000.sra.32188444, SRR768835.sra.13138622, SRR768835.sra.11666058, SRR768835.sra.14427516, SRR768835.sra.565413, SRR768835.sra.17372699 (> 10 RNA-Seq reads, others are not shown) and EST asmbl_2169.tomatov23pasa_pasa8.

The assembly of the variation site in *S. tuberosum* was supported by WGS reads. First, we found five WGS reads of *S. tuberosum* crossing the variation site in *S. tuberosum*: SRR307648.8944170, SRR307648.3412683, SRR307648.1519372, SRR307611.139971, and SRR307611.5927910. In addition, we found > 10 *S. tuberosum* WGS reads that matched the regions flanking the target variation site (1 kb at each side) in *S. lycopersicum* (SRR307587.1039929, SRR307587.1346404, SRR307587.2510704, SRR307587.2631231, SRR307587.2719574, SRR307598.1370881, SRR307598.2400506, SRR307598.3189858, SRR307598.3360774, and SRR307598.5122864, others are not shown) but no WGS reads of *S. tuberosum* that matched the target intron of *S. lycopersicum*, which indicates that the intron is absent in *S. tuberosum*.

## *Solyc01g086750.2*

*S. lycopersicum* CAGAACTGGATGAAAACATTTAAGATGCGTGAGCTGCAG---------------------------------------------------

*S. tuberosum*  CAGAACTGGATGAAAACATTTAAGATGCGTGAGCTGCAGgtataagttactcaagcctgatgatctctaatttcttgtaacaagattatc

*C. annuum*  CAGAACTGGATGAAAACATTTAAGATGCGTGAGCTGCAGgtataaattactcaagcctgatgacctctaattccttgttacaaccttatc

*S. lycopersicum* -----------------------------------------ATGAATGCACAAAAGCAGAAAGGTCAATCTCTGAATGTGGCTTCTGC

*S. tuberosum*  attcccttatgtgctttttgaaatatggaaatccttaacagATGAATGCGCAAAAGCAGAAAGGTCAATCTCTGAATGTGGCTTCTGC

*C. annuum*  attcctttatgttctttt-gcaatatggaaatccttaacagATGAATGTGCAAAAGCAGAAAGGTCAATCTTTGAATGTGGCTTCTGC

The *S. lycopersicum* gene *Solyc01g086750.2* has lost an intron. The active expression of the gene is supported by SRR567999.sra.17914021, SRR567999.sra.4676179, SRR567999.sra.13929817, SRR567999.sra.29493782, SRR567999.sra.37228191, SRR567999.sra.42598055, SRR567999.sra.41386493, SRR567999.sra.32139061, SRR567999.sra.20464663, and SRR567999.sra.8081166 (> 10 RNA-Seq reads, others are not shown).

The successful splicing of the target intron in *S. tuberosum* is supported by SRR184103.sra.1305954, SRR184103.sra.6786639, SRR184103.sra.4936490, SRR184103.sra.5086294, SRR184103.sra.1999365, SRR184104.sra.4439806, SRR866258.sra.9027335, SRR866258.sra.1883994, SRR866258.sra.6080894, SRR866259.sra.15214352 (> 10 RNA-Seq reads, others are not shown) and EST asmbl_1527.potatov3pasa_pasa0.

The successful splicing of the target intron in *C. annuum* is supported by SRR771929.10813798, SRR771929.11219895, SRR771929.11251710, SRR771929.13394144, SRR771929.13628800, SRR771929.16450280, SRR771929.26956822, SRR771929.27399501, SRR771929.28836371, and SRR771929.32749828 (> 10 RNA-Seq reads, others are not shown).

The assembly of the variation site in *S. lycopersicum* was supported by WGS reads. First, we found > 10 WGS reads of *S. lycopersicum* crossing the variation site in *S. lycopersicum*: ERR327647.30700662, ERR327647.20047053, ERR327646.208977, ERR327648.21330420, ERR327648.8805427, ERR327648.3178858, ERR327648.7868834, ERR327648.20953705, ERR327648.25616807, and ERR327648.30369586, others are not shown. In addition, we found > 10 *S. lycopersicum* WGS reads that matched the regions flanking the target variation site (1 kb at each side) in *S. tuberosum* (ERR327646.31199694, ERR327646.18717158, ERR327646.30388744, ERR327646.11144787, ERR327646.15963324, ERR327646.27220856, ERR327646.29686361, ERR327646.16447357, ERR327646.6393309, and ERR327646.14412852, others are not shown) but no WGS reads of *S. lycopersicum* that matched the target intron of *S. tuberosum*, which indicates that the intron is absent in *S. lycopersicum*.

#

# Imprecise Intron loss

## *PGSC0003DMG400000276*

*S. tuberosum*  GATGATGAGGATGAAGATGATAGCAGTACCAAGAAAAAG---------------------------------------------------

*S. lycopersicum* GATGATGAGGATGAAGATGATAGCAGTACCAAGAAAAAGgtacttacttccttgattagtggctaaataattatttggagagatggtcat

*S. melongena*  GATGAAGATGATGAAGATAATAACAATACCAAGAAAAAGgtacttcctttcttttttattcgctaaagttttatttg--gagatggccat

*S. tuberosum*  --------------------------------------------------------------gtatgaatcacgtactcatgtttcttca

*S. lycopersicum* gaatgctaacgttttcccccttgtttcaaatgttactacagTCATCATCTGCTGCTCGCAAGgtatgaatcacttgctcatgtttcttca

*S. melongena*  gaatgctaacgttttcccgcttttttcaaacatcactacagTCATCATCTGCTGCTCGCAAGgtatgaagaacgaatcc--ttttcttcg

*S. tuberosum*  atggaagttctttcatatgctttctcttttgtaataatagacatactgatttgcttatattcatgtcacagAGGATTGGTAGGGCACATG

*S. lycopersicum* aaacaagttctttcatgtgctttcgctttcacaatactagacatactgagttgtttatattcatgtcacagAAGATTGGTAGGGCACATG

*S. melongena*  ctgaaagttagttcatatgctttctcttccataaaattagacataccgagttgcttatatttatgtcgcagAGGATTGGTGGAGCACATG

The *S. tuberosum* gene *PGSC0003DMG400000276* lost an intron and a 21-bp-long downstream exon. The downstream intron is intact and has not lost any nucleotides; its successful splicing is supported by RNA-Seq reads SRR184103.sra.5219405, SRR184103.sra.4024466, SRR184103.sra.5198025, SRR184103.sra.5993881, SRR184103.sra.3737851, SRR184103.sra.663586, SRR184103.sra.4442482, SRR184103.sra.6316222, SRR184103.sra.785072 and SRR184103.sra.6507871 (> 10 RNA-Seq reads, others are not shown).

The successful splicing of the target intron in *S. lycopersicum* is supported by SRR568000.sra.14272634, SRR568000.sra.32217971, SRR568000.sra.10068574, SRR568000.sra.28554872, SRR568000.sra.10019954, SRR768863.sra.4184530, SRR768863.sra.21781785, SRR786507.sra.1972001, SRR786507.sra.934663, SRR768864.sra.3861563 (> 10 RNA-Seq reads, others are not shown) and EST asmbl_392.tomatov23pasa_pasa4.

The assembly of the variation site in *S. tuberosum* was supported by WGS reads. First, we found nine WGS reads of *S. tuberosum* crossing the variation site in *S. tuberosum*: SRR307648.16942931, SRR307648.4204856, SRR307648.15162300, SRR307648.12463400, SRR307648.18117605, SRR307648.7157612, SRR307648.16213302, SRR307648.15989747, and SRR307626.2649328. In addition, we found > 10 *S. tuberosum* WGS reads that matched the regions flanking the target variation site (1 kb at each side) in *S. lycopersicum* (SRR307587.1958810, SRR307587.2301420, SRR307587.4129610, SRR307587.4344412, SRR307587.475383, SRR307598.6522741, SRR307598.70466, SRR307605.1921017, SRR307605.2362182, and SRR307605.3648554, others are not shown) but no WGS reads of *S. tuberosum* that matched the target intron of *S. lycopersicum*, which confirmed the deletion in *S. tuberosum*.

## *Solyc08g007920.1*

*S. lycopersicum* tcctgatatatagGGTCCTACAAC------------------------------------------------------------------

*S. tuberosum*  tcttgatatatagGGTCCTACAACTCCTGATAAATTCGAGCAAATTATTCGTGCAAGGAGCATGAAAAATGATCAAATGTTTATGCTCAA

*C. annuum*  tctttatgcgtagGATCCTGCAACGCCAGAGAAATTCGAGCATGTTATCC------------TGAAGCACGATGAAATGCTTATGCTCAA

*S. lycopersicum* ------------------------------------------------------------------------------------------

*S. tuberosum*  Ggtatgaatgaccctaaagtatagtagaggataaagttaagatatttcgtatagcagaaaggtaaatttgtacatttccccccttatatc

*C. annuum*  GgtattaactattttaagatattgcagAGGATCAAGTTGGGAGGTGATTGATTCGGAAAAGGCA------CGAATCAGACCCCACGGATC

*S. lycopersicum* ------------------------------------------------------------------------------------------

*S. tuberosum*  tatctaacatatagtttgtattaatgcagAGGAATTCTTCAAACCATATAGGCAATGCCAATGCCAAACAGAGGAATTTATTTCATTCTT

*C. annuum*  -ATCTAAGATCCAGTTCA-ATTGATGATGAAAAGAGTGCCAAGCGATATAGACAATGTCACGTCTAAACAAAGGAAGCTATTCTACTCTT

*S. lycopersicum* ------------------------------------------------------------------------------------------

*S. tuberosum*  CAGAATTTAGCTTAAATTCAGAGCAATTCTGTTTTAGCTATGATGAAACATGTTACTCCTCTATTGATAATAGCCCACAACTTCACTCAA

*C. annuum*  CACATCTTAGCTTGAATTCTGACCAATATAGCTACAGCTTCGATGATGCTTGCTTTTGCACTGCTGACAATAGCCCTCAGTTTTACT---

*S. lycopersicum* ----ATCATCGAGATGTACTCGTTCGAGGACAGGACCATTTACGCCAACAAAGAGTAGTACACGAA

*S. tuberosum*  CAGCATCATCGAAATGTACTCGTTCGAGGACAGGACCATTTACGCCAACAAAGAGTAGTACACGAA

*C. annuum*  CGGCTTCATCAAAGGGCGGTAGTTCAAAAAGAGGACCGTTTACACCAACTAAGAGTGGTTCAAGAA

The *S. lycopersicum* gene *Solyc08g007920.1* lost a 118-bp-long intron, a 68-bp-long segment from an upstream exon and a 154-bp-long segment from a downstream exon. This deletion did not cause any frame shift events or inactivate the gene as supported by RNA-Seq reads SRR567999.sra.41972716, SRR567999.sra.41972716, SRR567999.sra.25828135, SRR567999.sra.8674299, SRR567999.sra.25828135, SRR567999.sra.4409168, SRR567999.sra.9113243, SRR567999.sra.8618055, SRR567999.sra.21309249, and SRR567999.sra.9113243 (> 10 RNA-Seq reads, others are not shown).

The successful splicing of the target intron in *S. tuberosum* is supported by SRR866258.sra.4833625, SRR866266.sra.9812980, SRR866266.sra.17755000, SRR866266.sra.13028572, SRR865383.sra.6847805, SRR866237.sra.4335401, SRR866237.sra.24934285, SRR866237.sra.1515607, SRR866237.sra.6606094, and SRR866237.sra.4772754 (> 10 RNA-Seq reads, others are not shown).

The successful splicing of the target intron in *C. annuum* is supported by SRR771940.16395967, SRR771940.28135893, SRR771940.16137344, SRR771940.8898474, SRR771940.22782584, SRR771940.11343704, SRR771940.27047023, SRR771940.21750991, SRR771940.18208268, and SRR771940.12903824 (> 10 RNA-Seq reads, others are not shown).

The assembly of the variation site in *S. lycopersicum* was supported by WGS reads. First, we found > 10 WGS reads of *S. lycopersicum* crossing the variation site in *S. lycopersicum*: ERR327646.7148743, ERR327646.9687924, ERR327646.32642529, ERR327646.1189044, ERR327646.9735179, ERR327646.23242056, ERR327646.4961790, ERR327646.7091701, ERR327646.4105396, and ERR327646.34001467, others are not shown. In addition, we found > 10 *S. lycopersicum* WGS reads that matched the regions flanking the target variation site (1 kb at each side) in *S. tuberosum* (ERR327646.31687330, ERR327646.4749846, ERR327646.26939959, ERR327646.8930916, ERR327646.15323276, ERR327646.4749846, ERR327646.18379568, ERR327646.4877363, ERR327646.16236380, and ERR327646.34124026, others are not shown) but no WGS reads of *S. lycopersicum* that matched the target intron of *S. tuberosum*, which confirmed the deletion in *S. lycopersicum*.

## *Solyc04g007270.2*

*S. lycopersicum* TAAGACTACTAACCCAAGCGGTAATTGCAAGGATGAATCAGTTGCTGGTGTGGGATTTGGGAAGAACCTGGCTCATGCTGTCTAAAGTGT

*S. tuberosum*  TAAGACTATGAATACAAGCGGTAATTGTAAGGATGAATCAGTTGCTGGTGTGGGATTAGGGAAGAACCTGGCTCATGTTGTCTAGAGCGT

*S. melongena*  CAAGTCTACAAATACAACTGGTAATTGCAAGGATGAATCAGTTGCTGGTGTGGGATTAGGGAAGAACCAGACTCATGCTGTCTAAAGCGC

*S. lycopersicum* GCTGGG------------------------------------------------------------------------------------

*S. tuberosum*  GCTGGGATTTTTAATAATGCTGTAATATgtaagtaatctacaaacttatataagc--agtttcttgggcagataacaaaatcagataaaa

*S. melongena*  TCTGGA-TTTTTAATAATGCTATAATAAgtaagtaacccataaacttatataggtagagtttttcgggtagagatcaagctcaaattcaa

*S. lycopersicum* ------------------------------------------------------------------------------------------

*S. tuberosum*  aaagaaacaga--ttttctgaagttgtctggcatttcgtaacttaatagatatcaatgctattgtactcctgtgaatgtagtaaaatggt

*S. melongena*  agataaaacagttttttctgaacttggctggcaatttttgtcaacgta-----cactgctattatacttctatggatgtagtaaaatagt

*S. lycopersicum* ------------------------------------------------------------------ATTATTTAATTGAGACCTTATCAA

*S. tuberosum*  cattgtcatttagatgtcaaactgaaacattttttcttcctgcctt---ctttgcagGTGGGTTCAATTATTGAATTGAGACCTGATCAA

*S. melongena*  cattgtcattcagacgtcgaactaaaatatttcttcttcctgtcttctgctttgcagGTGGATTCAACTATTGAATGGAGCTCTTATCAA

The *S. lycopersicum* gene *Solyc04g007270.2* lost a 204-bp-long intron, a 22-bp-long segment from an upstream exon and a 9-bp-long segment from a downstream exon. This deletion occurred in 3′-UTR and did not inactivate the gene as supported by RNA-Seq reads SRR567999.sra.11655909, SRR567999.sra.27999561, SRR567999.sra.15713852, SRR567999.sra.9392008, SRR567999.sra.7665759, SRR567999.sra.20227051, SRR567999.sra.14750879, SRR567999.sra.14221247, SRR567999.sra.14753761, and SRR567999.sra.13254655 (> 10 RNA-Seq reads, others are not shown).

The successful splicing of the target intron in *S. tuberosum* is supported by SRR184104.sra.6871089, SRR184104.sra.5248785, SRR184104.sra.4176053, SRR184104.sra.4861524, SRR184104.sra.4386106, SRR864485.sra.4649904, SRR864485.sra.7402028, SRR864485.sra.12592653, SRR864485.sra.12175127, and SRR864485.sra.8049457 (> 10 RNA-Seq reads, others are not shown).

The assembly of the variation site in *S. lycopersicum* was supported by WGS reads. First, we found > 10 WGS reads of *S. lycopersicum* crossing the variation site in *S. lycopersicum*: ERR418071.54084752, ERR418071.54084753, ERR418071.54084754, ERR418071.54084755, ERR418071.54084756, ERR418071.54084757, ERR418071.54084758, ERR418071.54084759, ERR418071.54084761, ERR418071.54084762, others are not shown. In addition, we found eight *S. lycopersicum* WGS reads that matched the regions flanking the target variation site (1 kb at each side) in *S. tuberosum* (ERR340383.87082589, ERR340383.87963130, ERR340383.122848827, ERR340383.73767882, ERR340383.121795509, ERR340383.106463425, ERR340383.75064668, and ERR340383.106741493) but no WGS reads of *S. lycopersicum* that matched the target intron of *S. tuberosum*, which confirmed the deletion in *S. lycopersicum*.

## *Solyc05g025890.1*

*S. lycopersicum* ACATGAGAGTATTTCAGCATTCTTGATAATGTCAGCAAGATCATC---------------------------------------------

*S. tuberosum*  ACATGAGAGTGTTTCTGCTTCCTGGATAATgtcagtaagctcatctatgattgggtatctatatatagtaaagatcaacatgtgtgttat

*N. benthamiana*  ACATCTGAACATTTCTGCTTCCTGGACAATgtcagtaagatcatctatgattgcttatatatataaagtaaagatcaacctct-tattat

*S. lycopersicum* -------------------GAATCTCAATTCCTCAAGCAAGGGGAAGAAA

*S. tuberosum*  tacttcattgcagGTCACTGAATCCCAATTCCTCAAGCACGGGGAAGAAA

*N. benthamiana*  tacgtta-agcagGTCATTGGATCAAAATTCCTCAGGCATAGGGAAGAAA

The *S. lycopersicum* gene *Solyc05g025890.1* lost an intron and a 6-bp-long segment from a downstream exon, and it simultaneously added a 15-bp-long segment to an upstream exon. This deletion did not cause any frame shift events or inactivate the gene as supported by RNA-Seq reads SRR567999.sra.5536337, SRR568000.sra.5103531, SRR768835.sra.7069657, SRR768835.sra.7972884, SRR768860.sra.9800795, SRR768860.sra.15211939, SRR768860.sra.2644669, SRR768862.sra.13759686, SRR768862.sra.14565171, and SRR768863.sra.349220 (> 10 RNA-Seq reads, others are not shown).

The successful splicing of the target intron in *S. tuberosum* is supported by SRR866258.sra.5343634, SRR866259.sra.14193683, SRR866259.sra.1854677, SRR866259.sra.8527606, SRR866275.sra.9070167, SRR866258.sra.12772706, SRR866258.sra.10756810, SRR866258.sra.7230968, SRR866275.sra.7212380, SRR864485.sra.2709386 (> 10 RNA-Seq reads, others are not shown) and EST asmbl_950.potatov3pasa_pasa6.

The successful splicing of the target intron in *N. benthamiana* is supported by SRR696988.sra.11722269, SRR696988.sra.11743316, SRR696988.sra.21344500, SRR696988.sra.22985329, SRR696988.sra.38041920, SRR696988.sra.42470792, SRR696988.sra.46339251, SRR696988.sra.4739287, SRR696992.sra.8381593, and SRR696988.sra.53144487 (> 10 RNA-Seq reads, others are not shown).

The assembly of the variation site in *S. lycopersicum* was supported by WGS reads. First, we found seven WGS reads of *S. lycopersicum* crossing the deletion site in *S. lycopersicum*: ERR327647.21662618, ERR327647.32759026, ERR327647.1440800, ERR327646.20836228, ERR327646.20908073, ERR327646.31927799, and ERR327647.26167014. In addition, we found six *S. lycopersicum* WGS reads that matched the regions flanking the target variation site (1 kb at each side) in *S. tuberosum* (SRR307587.1093413, SRR307587.3029773, SRR307587.3186372, SRR307587.3310308, SRR307587.3338431, SRR307587.637670, SRR307598.1042899, SRR307598.1234905, SRR307598.1748077, and SRR307598.1748077, others are not shown) but no WGS reads of *S. lycopersicum* that matched the region of *S. tuberosum* that are corresponding to the deletion of *S. lycopersicum* in position, which confirmed the deletion in *S. lycopersicum*.

## *Solyc06g054030.2*

*S. lycopersicum* CATCACCTTCCCCTATTCCA----------------------------------------------------------------------

*S. tuberosum*  CATCACCTTCCCCTGTTCCAGCTCCAGgtaccaaagttgctcaatttattaaattttgacaccatcctttcttgatttttgtttaaacaa

*C. annuum*  GTTCTGCTACCCCTCTTCCAGCACAAGgtaccaaaacaattcaatttatctaatcctg-cacc-tgtttttctaaatagtataggaagaa

*S. lycopersicum* ----------------------------------------------------------------------TCTCCTAGTCCTAAAG

*S. tuberosum*  gggaataagatgattaaatcttcatttggttacagGTCCAGCTAGCTTAAGACCAACTAGGTCACTTCCATCTCCTAGTCCTAAAG

*C. annuum*  aaaattaataaaatcaagtttttatttggctacagGTCCAGCTGCCTTAGCACCACCTAGATCACCTGCAGCTCCTAGCCCTGATG

The *S. lycopersicum* gene *Solyc06g054030.2* lost a 98-bp-long intron, a 7-bp-long segment from an upstream exon and a 35-bp-long segment from a downstream exon. This deletion did not cause any frame shift events or inactivate the gene as supported by RNA-Seq reads SRR768863.sra.8306301, SRR768863.sra.13456940, SRR768863.sra.16129845, SRR768863.sra.4024811, SRR768863.sra.7265769, SRR768863.sra.9476275, and SRR768863.sra.10885243.

The successful splicing of the target intron in *S. tuberosum* is supported by SRR184104.sra.4205728, SRR184104.sra.1756607, SRR184104.sra.4695357, SRR184104.sra.3536007, SRR184104.sra.4821393, SRR866266.sra.15471510, SRR866266.sra.9651562, SRR866266.sra.7184775, SRR866266.sra.10504521, SRR866275.sra.12931257 (> 10 RNA-Seq reads, others are not shown) and EST asmbl_837.potatov3pasa_pasa7.

The successful splicing of the target intron in *C. annuum* is supported by SRR771941.10969781, SRR771941.18071427, SRR771941.29482808, SRR771941.30998899, SRR771941.7787084, SRR771953.4269776, SRR771953.933992, SRR771949.12386529, SRR771949.200090, and SRR771949.24576196 (> 10 RNA-Seq reads, others are not shown).

The assembly of the variation site in *S. lycopersicum* was supported by WGS reads. First, we found > 10 WGS reads of *S. lycopersicum* crossing the variation site in *S. lycopersicum*: ERR327647.3429950, ERR327646.1787247, ERR327646.8905670, ERR327646.16564988, ERR327646.24134899, ERR327648.30233233, ERR327648.16652757, ERR327648.28331680, ERR327648.27220110, and ERR327648.30951506, others are not shown. In addition, we found > 10 *S. lycopersicum* WGS reads that matched the regions flanking the target variation site (1 kb at each side) in *S. tuberosum* (ERR327646.10707582, ERR327646.18901332, ERR327646.3993515, ERR327646.3444577, ERR327646.28940495, ERR327648.6925608, ERR327648.2024821, ERR327649.32290688, ERR327649.30050059, and ERR327649.1870350, others are not shown) but no WGS reads of *S. lycopersicum* that matched the target intron of *S. tuberosum*, which confirmed the deletion in *S. lycopersicum*.

## *Solyc04g071660.2*

*S. lycopersicum* TAGCCTAAGTCATGAAATGGAACGTTATATTTGCAGTGGAGTGGAAAGAGATCTCAGTGAATCGG-------------------------

*S. tuberosum*  TAGCCTAAGTCATGAAATGCAATCTTATATTTGCAGTGGAGTGGAAAGAGATCACAGTGAATCTGAAGCCGGGTCTATTGGAGTCGACCA

*C. annuum*  TAGCAAGAATCATGAAATGGAAACTTCAATATGCAGTGGAGTGAAAAGAAATCTTGGTGGATCTGAAGCTGGGTTTGTTGGAGTCAACCG

*S. lycopersicum* ------------------------------------------------------------------------------------------

*S. tuberosum*  AGgtagtaaattatccattttagtcataatggatttacacaagctgagtgttcacacatgaaattagtttgtttctgtttaagcttcttg

*C. annuum*  AGGTGgtaaagtatccct----accatagtggatttacacatgctgaattttcaaacatgaagttagtttgtgtttgttaaagcttcttg

*S. lycopersicum* ------------------------------------------------------------------------------------------

*S. tuberosum*  tactaacatcaaagccttatttcttgtagCTCCTGAGACTTCGCATATCACTGCTTTCAACTTTGGTGATGAAAATGAACAAGgttagtc

*C. annuum*  ctcttacaccaaagccttgtttcttgtagCTCCTGAGACTTCACATATTACTCCTGTCAACTATGGTGATGAAAA---ACAAGgttagtt

*S. lycopersicum* ------------------------------------------------------------------------------------------

*S. tuberosum*  cagaactatctccctgactgtataaactaaatttgactgacttcatcttctatttgctgctttattgtgcattaatagtgttaattcctg

*C. annuum*  ctgagccatctctcagaatgtataaattaacaatgactctccctgtcttctatttgctgctttattgtgtattaatagctttaattcctg

*S. lycopersicum* ------------------------------------------------------------------------------------------

*S. tuberosum*  atgcgcagAAGATCAATTGATAATGCCTTTTGCTTCAGCCAACATTACCCAGTCTATGGATCAAATGGATGGAACTCCAAGGATGACAGA

*C. annuum*  ctatgcagAAGATCTATCAAAAATGATGTTTGATTCACCCATCATTACCCAGTCTCTGGACCAAATGGATGGAACATCAAGAAAGACAGC

*S. lycopersicum* ------------------------------------------------------------------------------------------

*S. tuberosum*  AAACAAGAAACTTTTTATGTCTGGCAATGTTCAAGGTCTCTCTCGTAACAAAGTCTTTCCAGTTGCACATCCAAGTGCTATGGATCTGGA

*C. annuum*  TGACAGTAAAAAACTTTTGTCTGGCAGTGTTCTAGGGCTCTCTCAGAACAAAGGTTTTCCAGTTTCACATCAGAGTTCTATGGATCTGGA

*S. lycopersicum* ------------------------------------------------------------------------------------------

*S. tuberosum*  AGAGCCTGAGAGCATAGATTCTAAACATTCTGAAGAACAGAATAAAGTTGAGAAGATCAAAGAAACTGTTGAGTGGATGAAAGAATCCAG

*C. annuum*  AGAGTCTGAGAGAATAGATTTGAAATATTCTGAA------------------------------ACTGCTGAGTGGATGAAAGAATCCAG

*S. lycopersicum* ------------------------------------------------------------------------------------------

*S. tuberosum*  TGCTGTTGAAGAAGAGAACAATGACGGTCAGGGTAATATGCTCTCTGCAACATCAGCTAAAAGCATGACTCCATTTCGCAAGGATGAGCT

*C. annuum*  AGCTCTTGAAAAGGAGAACAATGAAGGACAGGGTAATATGCAGTCTGCAACATCAGCTAAAAGCATGGCTCCAGTGTGGAGTGATGAGTT

*S. lycopersicum* ------------------------------------------------------------------------------------------

*S. tuberosum*  TTCTTCTCAAAAACCTAAGATCAAAGAAACTGCTGAGTGCATGAAAGAATCCAGTACTCTTGAAGAAGAGAACAATGAAGGTCAGTGTAA

*C. annuum*  TTCTTCTCAAGAACGTACGATCAAAGAAAATGCTGAACGCATGAAAGAAACATGTGCTCTTGAAGAAGAGTACTATGAAGATCAGGGTAG

*S. lycopersicum* ------------------------------------------------------------------------------------------

*S. tuberosum*  TATGTTGTCTGGAATGTCAGCTAAAAACATGACTCCATTGCAGAATAATGAGTTTTTCTCTCAAGACACTAAGAAAGCAGATGTTGCAAC

*C. annuum*  TATGCTGTCTGCTACATCAGCTAAAAGCATGACTCCGTTGTGGAGTGGTGAGTTTTCTTCTCAAGAACCTATGATCAAAGAAACTACTGA

*S. lycopersicum* ------------------------------------------------------------------------------------------

*S. tuberosum*  GTCAGGAGAAATGGTTGGAGACACAGAGATGACTACAAGACATGAAAGCTGTGTTGAGGAAATTGTTGACATAGAGGAACAAAAAATCGA

*C. annuum*  GTCATGATGGAATCCAGAGCTCTTGAAGAAGATAATGG----TGAAGGTCA-ATGTAATATGCTGTCTGGA-ACGTCAGCTAAAAAT--A

*S. lycopersicum* ------------------------------------------------------------------------------------------

*S. tuberosum*  GGAAACAGTGGCTGACAGGGATTTGTTGTTATTTTCTCCTCAAGAACCTAAGACAGCAGATGTTGCAATGTCAGGAGAAATGGTTAGAGA

*C. annuum*  TGACTCCATTGCGGAAAGATGA--------GTTTCCTTCTCAAGAATCTAAGACAGCAGATGTTATGATATCACGACAAATGGTTAGAGA

*S. lycopersicum* ------------------------------------------------------------------------------------------

*S. tuberosum*  CACAGAGGTGAATACAATACATGAAATCTGTTTTCATGAAATTGTTGACATAGAGGAACACAAAATTGAGGAAACAGTGGCTGACAGGAA

*C. annuum*  CACAGAGATGAATACAAGACATGAAAGCTGTGTTCAGGAAATTGTTGACATAGAGGAACACAAAATTGAGGAAACAGTAACTGACAGGGA

*S. lycopersicum* ------------------------------------------------------------------------------------------

*S. tuberosum*  TTTGTTGCTAAGAGGAGATGTCTCTAGAGGTCTTGAAGAAGATATTTATGTTGGAATTGCAAATGAAAGCACTTCTCCTGTTATTTCAGA

*C. annuum*  TTTGTTGCTAAGAG---ATTCCTCTAGAGGTCACGAAGTAAATATTTATGCTGGAATTGCAAAGGAAAACACTTCTCCTGTTATTTCAGT

*S. lycopersicum* -----------------------TTTCTGAACAACAAATGGTGCCGGAGATCAATC

*S. tuberosum*  GCCTACATGCGATGATATTGGATTTTCTGAACAGCAAATGGTGCCGGAGATCAATC

*C. annuum*  GCCTACGTGCGATGATTTTGGATTTTCTGAACAGCGAATGGTACCGGAGATCAATA

The *S. lycopersicum* gene *Solyc04g071660.2* lost two introns (117-bp-long and 105-bp-long), their internal exon (54-bp-long), a 27-bp-long segment from an upstream exon, and a 915-bp-long segment from a downstream exon. This deletion did not cause any frame shift events. The active expression of the gene is supported by SRR567999.sra.22482606, SRR567999.sra.41414674, SRR567999.sra.29572891, SRR567999.sra.42074657, SRR567999.sra.25214598, SRR567999.sra.33206078, SRR567999.sra.33681282, SRR567999.sra.34410307, SRR567999.sra.34723966, and SRR567999.sra.8938156 (> 10 RNA-Seq reads, others are not shown).

The successful splicing of the target intron in *S. tuberosum* is supported by SRR866268.sra.9497890, SRR866268.sra.11904111, SRR866268.sra.5889566, SRR866268.sra.4243804 (for the first intron), SRR866268.sra.11904111, SRR866268.sra.6586943, SRR866268.sra.6402190, SRR866268.sra.8407719 (for the second intron) and EST asmbl_1407.potatov3pasa_pasa5.

The successful splicing of the target intron in *C. annuum* is supported by SRR771940.25066852, SRR771940.8772574, SRR771929.17291240, SRR771929.23996108, SRR771953.9541502, SRR771953.22949553, SRR771953.27282933, SRR771953.32802689, SRR771949.14967619, SRR771949.25954794 (> 10 RNA-Seq reads, for the first intron), SRR771940.17366349, SRR771940.27260330, SRR771940.25579957, SRR771929.34733212, SRR771929.17291240, SRR771953.27282933, SRR771953.6040723, SRR771953.31784818, SRR771953.32802689, and SRR771953.9541502 (> 10 RNA-Seq reads, for the second intron).

The assembly of the variation site in *S. lycopersicum* was supported by WGS reads. First, we found ten WGS reads of *S. lycopersicum* crossing the variation site in *S. lycopersicum*: ERR327647.30000919, ERR327646.18459235, ERR327647.15516121, ERR327647.18714109, ERR327646.2061482, ERR327646.23245089, ERR327647.21517566, ERR327646.33907853, ERR327646.33180538, and ERR327647.25359001. In addition, we found > 10 *S. lycopersicum* WGS reads that matched the regions flanking the target variation site (1 kb at each side) in *S. tuberosum* (ERR327646.25962131, ERR327646.12427777, ERR327646.22825500, ERR327646.7104350, ERR327646.21607878, ERR327646.30546259, ERR327646.20865038, ERR327646.20277998, ERR327646.11778262, and ERR327646.2061482, others are not shown) but no WGS reads of *S. lycopersicum* that matched the target intron of *S. tuberosum*, which confirmed the deletion in *S. lycopersicum*.

##

# Exon deletion

## *Solyc09g016940.2*

*S. lycopersicum* ACAGTCATCTTTAGCGGAGAACGAGAAACACTGTAGGATGGATTCAGAGgtgagttaaggcctcctcttttagcaactgctgagccattt

*S. tuberosum*  ACAGTCATCTTTAGCCGAGAATGAGAAACACTGTAGGATGGATTCAGAGgtgagttaaggcctcctcttttagcagctgctgagctattt

*N. benthamiana*  ACAGTCATCTTTAGCAGAAAATGAGAAACACAGTAGGATGGATACAGAGgtgagttaaggcctcctttttgggcaacttttgaactgctt

*S. lycopersicum* tcagttatgggctcataattttatgataattgtattctcttcccctctctcttatctgactattgtataactcatgacaagtcattgctt

*S. tuberosum*  tcagttatgggctcatcattttatgataattgtattctcttcccctctctcttatctgtctattgtataactcatgacaagtcattgttt

*N. benthamiana*  ttagttatgggctcattatttgacgataattgtattcgctatctctctcttctatctttatattatttaactcatgacaagtaattgttt

*S. lycopersicum* tctctattttatggacag------------------------------------------------------------------------

*S. tuberosum*  tctctattttatggacagGCCACCTTTGAAGAGAAAATTAAACAATTACAGAAGGACAAAGTGTCACATATGCAAAAGGAGgtataatgc

*N. benthamiana*  tctctattttatggacagCCCACTTCTCAAGAGAAAATTAAACAATTACAGAAAGACAAGGATGCATATATGCAAAAAGAGgtacaa-gc

*S. lycopersicum* ----------------------gcctttaaatgagcttacatctgcatggaatcccatatataatttctatctacatttaaattaaaaca

*S. tuberosum*  tgtaaaaccttttgcttgtgtagcctttaaatgagcttacatctgcatggaatcacatatatattttctatctacattttaattgaaaca

*N. benthamiana*  tgtaaaatcttttacttgggtagtctttacctgagtttacattcatataaagtcccgtatatatttcctatctacattataattgaagca

*S. lycopersicum* tttaaaagttactgctgttttacagGCTATCTTCGAGGAGAAACTTAAACAGTTCTCATGGGAAAAAGATGCCAGTTTACTGA

*S. tuberosum*  tttaaaagttactgctgttttacagGCTATCTTCGAGGAGAAACTTAAACAGTTCTCACGGGAAAAAGATGCCAGTTTACTGA

*N. benthamiana*  tctaatactttcagctgttttacagGCTATTTCTGAGGAGAAACTTAAACAATTAGCAAAGGAAAAAGATGCCAATTTACTGA

The *S. lycopersicum* gene *Solyc09g016940.2* lost an internal exon and the 5′ end of a downstream intron. This deletion resulted in the fusion of the previous two introns. The successful splicing of the target intron in the gene is supported by SRR567999.sra.14930084, SRR567999.sra.33717181, SRR567999.sra.6852467, SRR567999.sra.41921240, SRR768863.sra.10470943, SRR568000.sra.36321047, SRR568000.sra.17471781, SRR568000.sra.17542884, SRR568000.sra.26108041, SRR568000.sra.3954762 (> 10 RNA-Seq reads, others are not shown) and ESTs asmbl_914.tomatov23pasa_pasa12.

The successful splicing of the target introns in the *S. tuberosum* gene *PGSC0003DMG400004043* is supported by SRR184103.sra.3731372, SRR184103.sra.3736307, SRR184103.sra.2623809, SRR184103.sra.4613439, SRR184104.sra.3518487, SRR184104.sra.1277818, SRR866258.sra.59139, SRR866258.sra.1755607, SRR866258.sra.15503945, SRR866259.sra.19049707 (for the first intron, > 10 RNA-Seq reads, others are not shown), SRR184103.sra.2512573, SRR184103.sra.135768, SRR184104.sra.5641435, SRR184104.sra.64170, SRR184104.sra.319796, SRR184104.sra.2579169, SRR184104.sra.6720058, SRR866266.sra.15101059, SRR866266.sra.17722097, SRR866268.sra.5235776 (for the second intron, > 10 RNA-Seq reads, others are not shown) and EST asmbl_702.potatov3pasa_pasa10.

The successful splicing of the target introns in the *N. benthamiana* gene *NbS00005506g0019* is supported by SRR696884.sra.4660991, SRR696884.sra.5980345, SRR696884.sra.7584212, SRR696884.sra.1409973, SRR696884.sra.5392643 (for the first intron, > 5 RNA-Seq reads, others are not shown), SRR696884.sra.3894704, SRR696884.sra.11786848, SRR696884.sra.14792928, SRR696884.sra.6350204, and SRR696884.sra.1409973 (for the second intron, > 5 RNA-Seq reads, others are not shown).

# De-exonizaion

## *PGSC0003DMG400004043*

*S. tuberosum*  GTTCTCACGGGAAAAAGATGCCAGTTTACTGAAGGAGGAGgtaatagaaactacacacaaaatttgcattgtaaaaacctgcaacttgct

*S. lycopersicum* GTTCTCATGGGAAAAAGATGCCAGTTTACTGA---AGGAGgtaatagaaagtacacacaa--tttgcattgtaaaaacctgcaacttgct

*N. benthamiana*  ATTAGCAAAGGAAAAAGATGCCAATTTACTGA---AGGAGgtaacaaaaactacgtacaaattttgcatcatagaa-cctataactttct

*S. tuberosum*  aactcataggccagtggctggtgcctaatcagtttttttaagattttcactcctaatcttgcggatttaccttgatgtgcagtctaactt

*S. lycopersicum* aactcataggccagtggctggtgcctaatcagtttttt-aagattttcactcctaatcttgcggatttaccttgatgtgcagTCTAACTT

*N. benthamiana*  aattcataggccaaagtttggtgcttaatcagttttaagatattctttccttctaacattatggatttgccttgatctgcagTCTAACCT

*S. tuberosum*  taacgagaggattaaacaacttcagaatgaaaataacagttatctgcataaagaggtattttagttctgcctctgtctgaaagcaggcac

*S. lycopersicum* GAACGAGAGGATTAAACAACTTCAGAATGAAAATAACAGTCATCTGCATAAAGAGgtattttagttctgcctctgtctgaaagcaggcac

*N. benthamiana*  TCAAGAGAGGATTAAACAACTACAGACTGAAAATAACAATCATATATATAAAGAGgtatttttgaactgtccctgtcccaaaatgggcac

*S. tuberosum*  agtcaaaaaacaaagtgattttgaccaaatattaagagtatcctctcattattcaaatgtggggaaaactgtgttgtacttcttgtgtgt

*S. lycopersicum* agtgaaaaaacaaagtgattctgaccaaatattaagagttccctctcatgattcaaatgtggggaaaactgtgttgtacttctcgtgtgt

*N. benthamiana*  attgaggaaaacaattgattttggactaaaatttagtgtatcttttcgtgactcaaatattggaacaactgtgtagtactttttatgtgt

*S. tuberosum*  tgttcgctttatgtagtttcattttcaaaaatgaattgttcaagtacggttaaatttcgaagtctgatggtttgccatgctgctacgtgc

*S. lycopersicum* tgttcgagttatgtagtttcattttcaaaaatatattgttcaagtacgaataaatttcgaagtctgatggtatgccatgctgctacgtgc

*N. benthamiana*  tgtttgaattatgcattcacattttcaaaattgaattattctagtccacttaaattttgaatactggtgaagtatcatgctgctacatgc

*S. tuberosum*  acagagtatgcaaaatcagtgctgtgttttttaaatgtcttgtcct-tttatcctatctattttgagttttacgctcttctgtgatccac

*S. lycopersicum* ------------------------------------gtcttgtcctatttatcctatctatctcgagttttacgctcttctttggtccac

*N. benthamiana*  aacaaataggttgcaaatcattgctgtgttgctaatcccttttcctatttattctatctactctgagttttatgctcttctgcaatctgc

*S. tuberosum*  agGCTAGTTTTGAGATGAAAATCATGCAATTGCAAGATGAAATTAG

*S. lycopersicum* agGCTAGTTTTGAGATGAAAATCATGCAATTGCAAGATCAAATTAG

*N. benthamiana*  agGCTAGCTTTGAGGAGAAAATCATGCAATCTCAAAAAGAAATTAG

An internal exon of the *S. tuberosum* gene *PGSC0003DMG400004043* was converted into an internal region of a larger intron. Transcriptome data indicate that the *S. tuberosum* gene *PGSC0003DMG400004043* has two splicing patterns at this site: one is comprised of two introns and an internal exon and the other is comprised of a long intron. In contrast, the orthologous sites of *S. lycopersicum* and *N. benthamiana* each have only a single splicing pattern, which consists of two introns and one internal exon, as indicated by transcriptome data. The alternative transcript that spliced out a long intron is thus a novel transcript that originated from de-exonization in *S. tuberosum*.

The splicing of the target region to one intron of the gene in *S. tuberosum* is supported by RNA-Seq reads SRR866268.sra.5366652, SRR866275.sra.3891670, SRR866275.sra.5364896, SRR866275.sra.3875762, SRR866275.sra.10041806, SRR866275.sra.473019, SRR184103.sra.1899901, SRR184103.sra.296718, SRR866258.sra.3171505, and SRR866258.sra.15015076 (> 10 RNA-Seq reads, others are not shown). The splicing of the target region to two introns and an exon in *S. tuberosum* is supported by RNA-Seq reads SRR866259.sra.19049707, SRR866259.sra.16818903, SRR866259.sra.13599052, SRR866259.sra.2951488, SRR866259.sra.17815137, SRR866259.sra.10283676, SRR866259.sra.1573833, SRR866275.sra.6762126, SRR866268.sra.10511846, SRR866268.sra.2350182, (for the first intron, > 10 RNA-Seq reads, others are not shown), SRR866259.sra.14167999, SRR866259.sra.10487620, SRR866259.sra.16672208, SRR866259.sra.9349642, SRR866268.sra.7740952, SRR866268.sra.3259740, SRR866268.sra.11592160, SRR866266.sra.1808484, SRR866266.sra.12932519, SRR866266.sra.104594 (for the second intron, > 10 RNA-Seq reads, others are not shown) and EST asmbl_702.potatov3pasa_pasa10.

The splicing of the target region of the *S. lycopersicum* gene *Solyc09g016940.2* is supported by reads RNA-Seq SRR567999.sra.5246851, SRR567999.sra.1540581, SRR567999.sra.29933015, SRR567999.sra.8662187, SRR567999.sra.1640375, SRR567999.sra.36135577, SRR567999.sra.41470623, SRR567999.sra.24719498, SRR567999.sra.21761964, SRR768835.sra.13553225 (for the first intron, > 10 RNA-Seq reads, others are not shown), SRR567999.sra.14941029, SRR567999.sra.19921238, SRR567999.sra.1380359, SRR567999.sra.26830017, SRR567999.sra.827821, SRR567999.sra.28500339, SRR567999.sra.42443970, SRR567999.sra.8399316, SRR768835.sra.9817791, SRR768835.sra.3968917 (for the second intron, > 10 RNA-Seq reads, others are not shown) and EST asmbl_914.tomatov23pasa_pasa12, asmbl_915.tomatov23pasa_pasa1.

The splicing of the target region of the *N. benthamiana* gene *NbS00005506g0019* is supported by RNA-Seq reads SRR696884.sra.4660991, SRR696884.sra.5980345, SRR696884.sra.7584212, SRR696884.sra.1409973, SRR696884.sra.5392643 (for the first intron, > 5 RNA-Seq reads, others are not shown), SRR696884.sra.3894704, SRR696884.sra.11786848, SRR696884.sra.14792928, SRR696884.sra.6350204, and SRR696884.sra.1409973 (for the second intron, > 5 RNA-Seq reads, others are not shown).

# Intronizaion

## *PGSC0003DMG400022716*

*S. tuberosum*  CTTCCATCATCTAGCAATGGATTTTTCATTTTTCGgtaatttcccgttgttcaaaccccattctgcaaatgatatggcatcaacagttgc

*S. lycopersicum* CTTCCATCATCTAGCAATGGATTTTTCATTTTTCGGTAATTTCCCGTGGTTCAAACCCCATTCTGCAAATGATATGGCATCAACAGTTGC

*N. benthamiana*  CTTCCACTATCTGGCAATGGATTTTTCATTTCTCAGTAATTTCCTGTCGTTCAAACCCCACTCTGCGAAGGATATGGCGTCAACTGTTGT

*S. tuberosum*  atctgctagcaccctaatgcaaacacctaaacaaaatgctcagtttgatgctaagttttggaaatggactgtgttttcagTTCTTCCTTG

*S. lycopersicum* ATCTACTAGCACCCTAATGCAAACACCTAAACAAAATGCTCAGTTT------------TGGAAATGGACTGTGTTTTCATTTCTTCCTTG

*N. benthamiana*  ATCTACTAGTACCCTAATAGAAGCACCTAACCAAAAGGCTCAGTTTGATGCTAAGTTTTGGAAATGGACCCTGTTTTTATTTGTTCCTTG

*S. tuberosum*  GGCTAAAGGAGCTGAGGGTAATATTCAAATGCCAACAACTGTCAACAAGAAACTGAAAAG

*S. lycopersicum* GGCTAAAGTAGCCGAGGGTAATATTCAAATGCCAACAACTGTCAACAAGAAACTGAAACG

*N. benthamiana*  GGCCAAAGGATCCGAGGGTAATATTCAGATGCCAACAACTGTCAACAAGAAGCTGAAAAG

The *S. tuberosum* gene *PGSC0003DMG400022716* has gained an intron. A T to G conversion has created a new 3′ splicing site (ag). The successful splicing of the target intron is supported by SRR184104.sra.6934631, SRR184104.sra.5007336, SRR184104.sra.9356988, SRR184104.sra.5960384, SRR866266.sra.1787205, SRR866266.sra.5646250, SRR866266.sra.13771529, SRR865383.sra.12167034, SRR866243.sra.12070895, and SRR866243.sra.8873657.

The target region of the *S. lycopersicum* gene *Solyc01g008780.2* is not spliced, which is supported by RNA-Seq reads SRR567999.sra.40941445, SRR567999.sra.8062218, SRR567999.sra.22108235, SRR567999.sra.37241996, SRR567999.sra.28233552, SRR786507.sra.3336287, SRR768863.sra.421010, SRR786507.sra.1983500, SRR768860.sra.3824411, SRR768860.sra.10473676 (> 10 RNA-Seq reads, others are not shown) and EST asmbl_510.tomatov23pasa_pasa1.

The target region of the *N. benthamiana* gene *NbS00013515g0018* is not spliced, which is supported by RNA-Seq reads SRR696884.sra.13323143, SRR696884.sra.2165169, SRR696884.sra.11778978, SRR696884.sra.13323143, and SRR696884.sra.408863 (> 5 RNA-Seq reads, others are not shown).

## *Solyc06g068980.2*

*S. lycopersicum* AGTGCAGGAAATGAATCTCCCTCTTCTGGTAATCCAGAACTGCAGgtaaaagcggaaaaagaatcttataataccttccttgattttaca

*S. tuberosum*  AGTGCAGGAAATGAATCTCCCTCTTCTGGTAATTCAGAACTGCAGGTAAAAGCGGAGAAAGAATTTTATAATACCTTCCATGATTTTACA

*N. benthamiana*  ACTGCAGGAAATGAATTTCCCTCTTCTAGTAATTTGGAACAGCAGATAAAAGCAGAAAAAGGAGGTTATAATACTTTCAATGATTTTACG

*S. lycopersicum* aaggagcaaggacagGAAACTTCTTCTAGAGCTGGGCATGCAAGATCTGCTTTTACACATGCAAGATCTCCTTCG

*S. tuberosum*  AAGGAGCAAGGACAGGAAACATCTTCTAGAGCTGGGCATGCAAGATCTGCTTTTACACATGCAAGATCTCCTTCC

*N. benthamiana*  AAGGAGCAGGGGCAAGAAACTTCTTCGAGAGCTGGGCATGCAAGATCTCCTTTTACACATGCAAGATCTCCTTCG

An internal exonic segment of the *S. lycopersicum* gene *Solyc06g068980.2* was converted into an intron. Although the GT..AG motifs are also present in *S. tuberosum*, transcriptome data do not support the splicing of this segment in *S. tuberosum*. We suspect that cryptic splicing signals have already been presented in *S. tuberosum* and *S. lycopersicum*, but only in *S. lycopersicum* they were only activated as active signals which co-occurred through nucleotide substitutions at other sites within the segment or at sites flanking the segment.

The successful splicing of the target intron is supported by SRR567999.sra.37011766, SRR567999.sra.33204644, SRR567999.sra.43040883, SRR567999.sra.17277377, SRR567999.sra.38138493 SRR567999.sra.29482373, SRR567999.sra.38398571, SRR768863.sra.11262011, SRR768863.sra.17548625, SRR768864.sra.2238898 (> 10 RNA-Seq reads, others are not shown) and EST asmbl_1979.tomatov23pasa_pasa9.

The target region of the *S. tuberosum* gene *PGSC0003DMG401028784* is not spliced, which is supported by RNA-Seq reads RR184103.sra.588491, SRR184103.sra.4672937, SRR184103.sra.3245017, SRR184103.sra.2871859, SRR184103.sra.2366303, SRR866259.sra.4588424, SRR866259.sra.12913319, SRR866275.sra.3750101, SRR866275.sra.10193511, SRR866275.sra.7574554 (> 10 RNA-Seq reads, others are not shown) and EST asmbl_1613.potatov3pasa_pasa7.

The target region of the *N. benthamiana* gene *NbS00008935g0001* is not spliced, which is supported by RNA-Seq reads SRR696884.sra.837660, SRR696884.sra.11162639, SRR696884.sra.11656699, SRR696884.sra.10468274, and SRR696884.sra.5247446 (> 5 RNA-Seq reads, others are not shown).

# Exonization

## *Solyc10g005290.2*

*S. lycopersicum* TTCTTCAACCTTGATGGAGCCCGTCCTCAGAAAGTTGAGCCTCTTTTGATAAGCAAGgtatagctcatgttcattgaagcactttttagg

*S. tuberosum*  TTCTTCAACCTTGATGGAGCCCGTCCTCAGAAAGTTGAGCCTCTTTTGATAAGCAAGgtatagcacatgttcattgaagctctttttagg

*C. annuum*  TTCTTCAACCTTGATGGTGCCCGTCCTCAGAAAGTTGAGCCTCTTTTTATAAGCAAGgtatagcacatgtcctctgaacctctctttaag

*S. lycopersicum* caacattggaaaggaacaactgagaagatatcttgtaattttacacgaacaattttgcaatatttttcgcagTTCATAAGTAACACAACA

*S. tuberosum*  caac--tggaaaggaataactgagaggatagcttgtaattttacacgaacaattttgcaatatatttcccggttcataagtaaaacaaca

*C. annuum*  caacattgtcaaggaataactgagaggatagcttgtaattttgaatgaacaattttgcaaaatatttcaaggttgatgaataaaaaaaga

*S. lycopersicum* TATTGTATCTGGAATTACGGGGTCTGTGGAGGGTGGCGTgtatgcagactttacccttaccttatagagatagagaggcaaactagtatt

*S. tuberosum*  tattgtatctgggattatggggtctgcggagggtggtgtgtgtgcagaccttacccctaccttgtagagatagagaggcaaactagtatt

*C. annuum*  tattgtatccaggattatggggtctggggagggtggtgtgtacgcagaccttacccctacctcgtagagatagagagccaaactagtatt

*S. lycopersicum* ccagttcataattacattgaaacatgtacagGTAAATACAGTTCTGCAATTGGCCCTTGTTGCTGCAGCTCTCCTTCAA

*S. tuberosum*  ccagttcataactatattgaaacatgtacagGTAAATACAGTTCTGCAATTGGCCCTTGTTGCTGCAGCTCTCCTTCAA

*C. annuum*  ccagttcataacaatattccaatatgtacagGTAAATACAGTTTTGCAATTGGCCCTCGTTGCTGCAGCTCTCCTTCAA

An internal region of a large intron has been converted into an internal exon by creating a 3′ splicing site (AG, a G to A transition) upstream of the region. Transcriptome data indicate that the *S. lycopersicum* gene *Solyc10g005290.2* has two splicing patterns at this site: one is comprised of two introns and an internal exon and the other is comprised of a long intron. In contrast, orthologous sites in *S. tuberosum* and *C. annuum* each have only one splicing pattern, which is comprised of a long intron and was indicated by transcriptome data. The alternative transcript with the novel exon in *S. lycopersicum* therefore originated from exonization. The splicing of the target region to two introns and one exon in *S. lycopersicum* is supported by RNA-Seq reads SRR768851.sra.23422940, SRR768851.sra.12275388, SRR768851.sra.10420727, SRR768851.sra.22477511, SRR568000.sra.37163170, SRR568000.sra.20876151, SRR568000.sra.10359382, SRR568000.sra.8040871, SRR568000.sra.34405870 (for the first intron) and SRR768851.sra.26485600, SRR568000.sra.34405870, SRR568000.sra.29353594, SRR568000.sra.33349367, SRR568000.sra.28153456 (for the second intron) and EST asmbl_40.tomatov23pasa_pasa2. The splicing of the target region to one intron is supported by RNA-Seq reads SRR768835.sra.13249134, SRR768835.sra.4864019, SRR768835.sra.9595667, SRR768835.sra.3055780, SRR768835.sra.12301595, SRR768835.sra.5827841, SRR567999.sra.30051348, SRR567999.sra.32363153, SRR567999.sra.19736281, SRR567999.sra.33159056 (> 10 RNA-Seq reads, others are not shown) and EST asmbl_39.tomatov23pasa_pasa2.

The successful splicing of the target intron in the *S. tuberosum* gene *PGSC0003DMG400011258* is supported by SRR866266.sra.15838966, SRR866266.sra.3457269, SRR866266.sra.12250385, SRR866266.sra.13284119, SRR866266.sra.5271634, SRR866258.sra.4626379, SRR866258.sra.6068142, SRR866258.sra.5225757, SRR866258.sra.6753305, SRR866258.sra.5988371 (> 10 RNA-Seq reads, others are not shown) and EST asmbl_160.potatov3pasa_pasa11.

The successful splicing of the target intron in the *C. annuum* gene *Capana10g000595* is supported by SRR771940.1399910, SRR771940.8297974, SRR771940.440897, SRR771940.27361809, SRR771940.20139148, SRR771940.10916965, SRR771940.6845517, SRR771940.10028084, SRR771940.28035043, and SRR771940.16138870 (> 10 RNA-Seq reads, others are not shown).

Data S2. Differences at deletion positions among transcripts.

In the orthologous gene that corresponded to the IIL gene, *Solyc05g025890.1*, the transcripts were different at the position of intron variation. We used FPKM values, which are outputs of Cufflinks [18], to represent the expression levels of transcripts. The positions of intron variations are enclosed in black rectangular boxes.


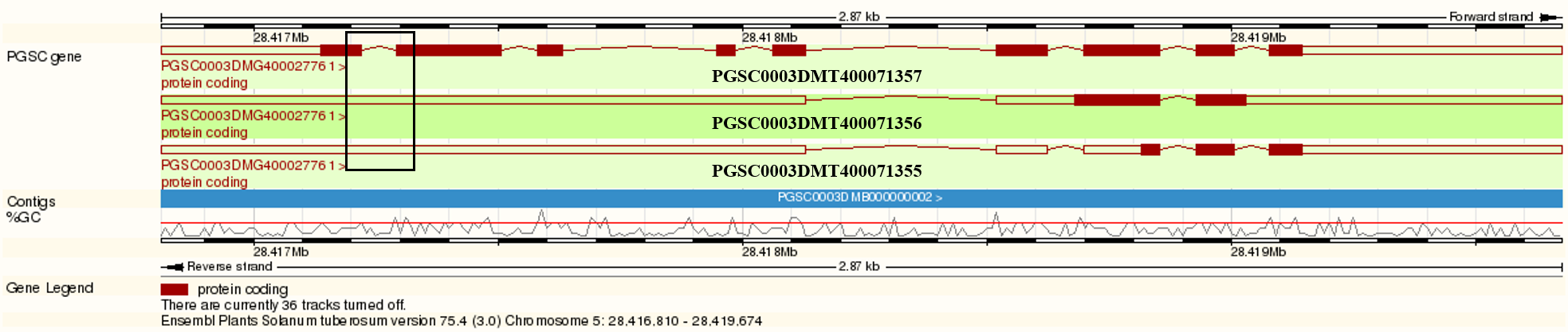


The lost intron of the tomato gene *Solyc05g025890.1* was annotated in the principle transcript (*PGSC0003DMT400071357*) of the potato orthologous gene *PGSC0003DMG400027761*. The potato gene *PGSC0003DMG400027761* has three alternative transcripts *PGSC0003DMT400071357*, *PGSC0003DMT400071356*, and *PGSC0003DMT400071355*, with FPKM values of 8.475, 0.843, and 0.00011, respectively. Based on its FPKM value, we identified *PGSC0003DMT400071357* as the principle transcript. Therefore, this deletion in the tomato gene *Solyc05g025890.1* can be regarded as an intron loss.

This image was downloaded from <http://plants.ensembl.org/Solanum_tuberosum/Location/View?db=core;g=PGSC0003DMG400027761;r=5:28416810-28419674>.

1. Bombarely A, Rosli HG, Vrebalov J, Moffett P, Mueller LA, Martin GB. A draft genome sequence of *Nicotiana benthamiana* to enhance molecular plant-microbe biology research. Mol Plant Microbe Interact. 2012;25(12):1523-30.

2. Bombarely A, Menda N, Tecle IY, Buels RM, Strickler S, Fischer-York T et al. The Sol Genomics Network (solgenomics.net): growing tomatoes using Perl. Nucleic Acids Res. 2011;39:D1149-D55.

3. Qin C, Yu C, Shen Y, Fang X, Chen L, Min J et al. Whole-genome sequencing of cultivated and wild peppers provides insights into *Capsicum* domestication and specialization. Proc Natl Acad Sci USA. 2014;111(14):5135-40.

4. Phytozome. http://www.phytozome.net/.

5. NCBI. ftp://ftp.ncbi.nih.gov/genomes/.

6. The Sequence Read Archive of NCBI. http://www.ncbi.nlm.nih.gov/sra/.

7. SynMap: Whole Genome Synteny. http://genomevolution.org/CoGe/SynMap.pl.

8. Larkin MA, Blackshields G, Brown NP, Chenna R, McGettigan PA, McWilliam H et al. Clustal W and clustal X version 2.0. Bioinformatics. 2007;23(21):2947-8.

9. Edgar R. MUSCLE: a multiple sequence alignment method with reduced time and space complexity. BMC Bioinformatics. 2004;5(1):113.

10. Roy SW, Penny D. Patterns of intron loss and gain in plants: Intron loss-dominated evolution and genome-wide comparison of *O. sativa* and *A. thaliana*. Mol Biol Evol. 2007;24(1):171-81.

11. Csuros M, Rogozin IB, Koonin EV. A detailed history of intron-rich eukaryotic ancestors inferred from a global survey of 100 complete genomes. PLoS Comput Biol. 2011;7(9):e1002150.

12. Rogozin I, Carmel L, Csuros M, Koonin E. Origin and evolution of spliceosomal introns. Biol Direct. 2012;7(1):11.

13. Wolf YI, Koonin EV. Genome reduction as the dominant mode of evolution. Bioessays. 2013;35(9):829-37.

14. Sarkinen T, Bohs L, Olmstead RG, Knapp S. A phylogenetic framework for evolutionary study of the nightshades (Solanaceae): a dated 1000-tip tree. BMC Evol Biol. 2013;13:214.

15. Kim D, Pertea G, Trapnell C, Pimentel H, Kelley R, Salzberg SL. TopHat2: accurate alignment of transcriptomes in the presence of insertions, deletions and gene fusions. Genome Biol. 2013;14(4):R36.

16. Li H, Durbin R. Fast and accurate short read alignment with Burrows-Wheeler transform. Bioinformatics. 2009;25(14):1754-60.

17. Rodriguez JM, Maietta P, Ezkurdia I, Pietrelli A, Wesselink J-J, Lopez G et al. APPRIS: annotation of principal and alternative splice isoforms. Nucleic Acids Res. 2013;41(D1):D110-D7.

18. Trapnell C, Williams BA, Pertea G, Mortazavi A, Kwan G, van Baren MJ et al. Transcript assembly and quantification by RNA-Seq reveals unannotated transcripts and isoform switching during cell differentiation. Nat Biotechnol. 2010;28(5):511-5.
