# Supplementary material for: Imprecise intron losses are less frequent than precise intron losses but are not rare in plants
Source: Biol Direct. 2015 May 27;10:24. doi: 10.1186/s13062-015-0056-7 (PMC4443532; doi:10.1186/s13062-015-0056-7)
Supplement: Supplementary file 3 — Details of intron variations in Arabidopsis thaliana. A complete list of supplementary materials, methods, and figures is given in this file. [file 13062_2015_56_MOESM3_ESM.docx]

Additional File 3

**Imprecise intron losses are less frequent than precise intron losses but are not rare in plants**

Ming-Yue Ma, Tao Zhu, Xue-Nan Li, Xin-Ran Lan, Heng-Yuan Liu, Yu-Fei Yang, and Deng-Ke Niu

**Materials and methods**

We downloaded the genome sequences and annotation files of *A. thaliana* Col-0 (TAIR release 10), *A.* *lyrata* (JGI release v1.0), *B.* *rapa* (version 1.2), *C.* *rubella* (JGI release v1.0), *O. sativa* (MSU Release 7.0), and *T.* *halophila* (JGI release v1.0) from Phytozome (version 9.1) [1]. The genome sequences of 180 lines of *A. thaliana* from Sweden were downloaded from the Gregor Mendel Institute of Molecular Plant Biology [2, 3]. The SAR files of the genomes and transcriptomes of *A. lyrata* (SRP004429 and SRP020356), *A. thaliana* (SRP026222, SRP009136 and SRP010096), *C. rubella* (SRP020007), and *T. halophila* (SRP012233) were retrieved from the Sequence Read Archive of NCBI [4].

The methods used to identify intron losses in *A. thaliana* are similar to those that were used in potato and tomato (see Additional File 2).

By comparing the gene structures of 16,266 orthologs between *A. thaliana* and *A. lyrata*, we found 206 putative imprecise intron variation sites. Manual inspection of these sites excluded 40 sites that were located in unreliable alignment regions. After the remaining 166 sites were polarized using *C. rubella*, *Thellungiella halophila*, and *Brassica rapa* as outgroups (Additional File 1, Data S3), 69 putative cases of IILs in *A. thaliana* were identified. Using the RNA-Seq data of *A. lyrata*, *C. rubella*, *T. halophila*, and *B. rapa*, we confirmed that 18 introns that were lost in *A. thaliana* could be successfully spliced (supported by ≥ 3 supporting RNA-Seq reads) in at least two of the other four species. By consulting the WGS sequencing data of the reference strain (Columbia-0) of *A. thaliana* (SRR960120), we found that one putative IIL that was located in the second exon of gene *AT4G16590* might have been due to assembly error. The remaining 17 genes are actively expressed in *A. thaliana*, as indicated by RNA-Seq data (see Additional File 1, Data S3 for the corresponding multiple species alignments and supporting RNA-Seq reads).

Close examination of 114 cases of intron loss from *A. thaliana* that had been reported in a previous paper [5] revealed that 104 cases in 98 genes were PILs, two cases were IILs, and eight cases lacked the support of a sufficient number of RNA-Seq reads. These two cases of IILs are included in the above 17-case dataset of IILs.


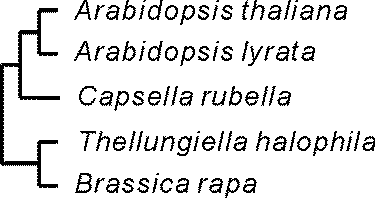


The phylogenetic tree used to distinguish intron loss and gain in *A. thaliana*. The tree was adapted from Phytozome (version 9.1, http://www.phytozome.net/). Its branches were not scaled according to the substitution rates. Standard parsimony was used to define intron losses. Full lists of the presence, absence and uncertainty of introns in the orthologous genes of the five species are shown in the following table.

Presence and absence of introns in *A. thaliana* and outgroups.

| *A. thaliana* |  | *A. lyrata* |  | *C. rubella* |  | *B. rapa* |  | *T. halophila* |  |
| --- | --- | --- | --- | --- | --- | --- | --- | --- | --- |
| AT1G03780 | - | fgenesh2_kg.1__305__AT1G03780.2 | + | Carubv10008388m | + | Bra015263 | + | Thhalv10006864m | + |
| AT1G13730 | - | fgenesh2_kg.1__1486__AT1G13730.1 | + | Carubv10011148m | + | Bra019707 | - | Thhalv10007774m | + |
| AT1G17680 | - | fgenesh2_kg.1__1931__AT1G17680.1 | + | Carubv10008264m | + | Bra025955 | + | Thhalv10006758m | + |
| AT1G48175 | - | fgenesh1_pm.C_scaffold_1003032 | + | Carubv10009558m | + | Bra014105 | + | Thhalv10001035m | + |
| AT1G54680 | - | fgenesh1_pm.C_scaffold_1003642 | + | Carubv10020727m | + | Bra021330 | + | Thhalv10023619m | + |
| AT2G39240 | - | fgenesh1_pm.C_scaffold_4001672 | + | Carubv10022885m | + | - | ? | - | ? |
| AT2G47910 | - | fgenesh2_kg.4__3042__AT2G47910.1 | + | Carubv10023930m | + | Bra021447 | + | Thhalv10001598m | + |
| AT3G07160 | - | fgenesh1_pg.C_scaffold_3000671 | + | Carubv10022498m | + | Bra029628 | + | Thhalv10019878m | + |
| AT3G16940 | - | fgenesh1_pm.C_scaffold_3001474 | + | Carubv10012963m | + | Bra022188 | + | Thhalv10020047m | + |
| AT3G17330 | - | Al_scaffold_0003_1823 | + | Carubv10013212m | + | - | ? | - | ? |
| AT3G27670 | - | fgenesh2_kg.5__547__AT3G27670.1 | + | Carubv10016560m | + | Bra025295 | + | Thhalv10003508m | + |
| AT3G54170 | - | Al_scaffold_0005_2321 | + | Carubv10017589m | + | Bra007052 | + | Thhalv10010523m | + |
| AT3G63400 | - | fgenesh1_pm.C_scaffold_5002308 | + | Carubv10016960m | + | Bra040447 | + | Thhalv10006033m | + |
| AT4G23040 | - | fgenesh2_kg.7__1994__AT4G23040.1 | + | Carubv10004557m | + | - | ? | - | ? |
| AT4G38530 | - | fgenesh2_kg.7__50__AT4G38530.1 | + | Carubv10007023m | + | Bra033567 | + | Thhalv10024782m | + |
| AT5G24470 | - | scaffold_602544.1 | + | Carubv10000600m | + | Bra009768 | - | Thhalv10003894m | + |
| AT5G60790 | - | fgenesh1_pm.C_scaffold_8001672 | + | Carubv10026078m | + | Bra023575 | + | Thhalv10012937m | + |

“+” indicates the presence of an intron at the corresponding position, “-” indicates the absence of intron at the corresponding position and “?” indicates that no confident result was observed.

Aligned sequences showing imprecise intron losses in *A. thaliana*. Intronic sequences are presented in lowercase and exonic sequences are in uppercase. Indels that occurred before the divergence of *A. thaliana* and *A. lyrata* and those that occurred in outgroup species (*e.g*., *C. rubella*) are not relevant to our study and would result in confusion if their alignments were presented. Therefore, these indels were excluded from the following alignments. Alignments containing intact sequences are available upon request ([dengkeniu@hotmail.com](mailto:dengkeniu@hotmail.com), [dkniu@bnu.edu.cn](mailto:dkniu@bnu.edu.cn)). The reads that supported the annotated introns are presented below each alignment. In cases where there are > 10 reads, only the names of 10 reads are listed.

[*AT1G03780* 4](#_Toc2127517160)

[*AT1G13730* 5](#_Toc1020359429)

[*AT1G17680* 6](#_Toc861383845)

[AT1G48175 7](#_Toc2116165245)

[AT1G54680 8](#_Toc848725507)

[AT2G39240 9](#_Toc1288982573)

[AT2G47910 10](#_Toc149418253)

[AT3G07160 11](#_Toc77045814)

[AT3G16940 12](#_Toc371066318)

[AT3G17330 13](#_Toc1589188181)

[AT3G27670 14](#_Toc1783563228)

[AT3G54170 15](#_Toc91912790)

[AT3G63400 16](#_Toc363933244)

[AT4G23040 17](#_Toc1172016803)

[AT4G38530 18](#_Toc996592524)

[AT5G24470 19](#_Toc1896395094)

[AT5G60790 20](#_Toc1902274340)

# ***AT1G03780***

*A. thaliana* GTTTATGTTCGTGAGCAAATTGCACCTTTTGTTTCAACTGCTGAATTGATGAAGAAGTTCCAAACAAGCACGCGAGACCTAT--------

*A. lyrata*  GTTTATGTCCGTGAGCAAACTGCACCTTTTGTTTCAACTGCTGAATTGATGAAGAAGTTCCAAACAAGCACCCGAGACCTGTCATTGCCT

*C. rubella*  GTTTATGTCCGTGAGAAAATTGCACCTTTTGTTTCAACTGCTGAACTGATGAAGAAGTTCCAAACAAGCACCCGAGACTTGTCATTGCCT

*A. thaliana* ------------------------------------------------------------------------------------------

*A. lyrata*  CACGGAAACACTTCTCTTCCACAGgtaataaacaattgaaactctctaaatccatttcatttcctgggaatctctttgattaaattccag

*C. rubella*  CACGCCAACACTTCTCTTCTGCAGgtaataaagaatcgaaactctccacttccttttacttctcttggaatctctttgactaaattccag

*A. thaliana* ----------------TCGTCCAGAATCGACCTAAGCTGACACTGACAAGACCAAAGGAACCCGAATTTGTGACGTCCCAACGAGCTCGT

*A. lyrata*  gatggtgctgttttctttgtccagAATCGACCTAAGCTGACACTGACAAGACCAAAGGAACCCGAATTTGTGACATCCCAACGAGCTCGT

*C. rubella*  gatggtactgttttttttgtccagAATCGACCTAAGCTGACACTGACAAGACCGAAGGAGCCTGAATTTGTGACATCTCAACGAGCTCGT

The *A. thaliana* gene *AT1G03780* lost an intron and a 33-bp-long segment from an upstream exon, and it simultaneously added a 9-bp-long segment to a downstream exon. This deletion did not cause any frame shift events. The activate expression of the gene is supported by SRR360152.11587894, SRR360152.22054825, SRR360152.27246684, SRR360152.30029433, SRR360152.4783136, SRR360152.15127827, SRR360152.28631427, SRR360152.28741441, SRR360152.17227103, and SRR360152.30250230 (>10 RNA-Seq reads, others are not shown).

The successful splicing of the target region of *A. lyrata* is supported by SRR800645.4147240, SRR800645.3655985, and SRR800644.4319061.

The successful splicing of the target region of *C. rubella* is supported by SRR797555.14681989, SRR797555.27016211, SRR797555.5325959, SRR797558.10950219, SRR797558.3079003, SRR797558.7222928, SRR797556.17788692, SRR797558.29808726, SRR797555.43582650, and SRR797555.9101432 (>10 RNA-Seq reads, others are not shown).

The assembly of the variation site in *A. thaliana* was supported by WGS reads. First, we found >10 WGS reads of *A. thaliana* crossing the deletion site in *A. thaliana*: SRR960120.27201549, SRR960120.1763394, SRR960120.7361598, SRR960120.20894425, SRR960120.5783395, SRR960120.5271676, SRR960120.25447287, SRR960120.869560, SRR960120.5284851 and SRR960120.28840635, others are not shown. In addition, we found >10 *A. thaliana* WGS reads that matched the regions flanking the target variation site (1 kb at each side) in *A. lyrata* (SRR960120.27201549, SRR960120.1763394, SRR960120.7361598, SRR960120.20894425, SRR960120.5783395, SRR960120.5271676, SRR960120.25447287, SRR960120.869560, SRR960120.5284851 and SRR960120.28840635, others are not shown) but no WGS reads of *A. thaliana* that matched the region of *A. lyrata* that are corresponding to the deletion of *A. thaliana* in position, which confirmed the deletion in *A. thaliana.*

# ***AT1G13730***

*A. thaliana* TTCTCAACGATGTCTTTAGGTATGTTGCTGATGAGATTGTTGAACCAGAAGCTAACAAGAAAGAAGTCGAGGAGGTTATTCCTCAAGTGG

*A. lyrata*  TGCTGAACGATGTCTTTAGGTATGTCGCTGATGAGATTGTTGAACCAGAAGCTAACAAGAAGGAGGTCGAGGAGGTGATTCCTCAAGTGG

*C. rubella*  TGCTGAACGATGTCTTTAGGTATGTCGCTGATGAGGTTGTTGAACCAGAAGCTAACAAGAAAGATGTCGAGGAGGTGATTCCTCAAGAGG

*A. thaliana* TCCAACCAA---------------------------------------------------------------------------------

*A. lyrata*  TCCAATCAACAGTCACTGTTTTGGgtgagtattggctaatgattttttgtcaatcagaaaaattagaatgacatttcggatttcataaga

*C. rubella*  TTCAGTCAACAGTCACTGTAgtgggtaagtattgg----------tttgtcaatcaagaaaatcaaatttacatttcggatttcataaca

*A. thaliana* ------------------CAGAGCAGGTAGATGAAGTTGCAGAGCCGGTTACTATCCCTACCCAACAACCCGAGGCGAAACAGACAACCG

*A. lyrata*  atattcggttttgatcagCAGAGCCGGCGAATGAAGTTGCAGAGCCAGTTACTATCCCTAGTCAACAGCCCGCAGCGAAACACACTACTG

*C. rubella*  ata-tcatttatgatcagcagagCCAAGAACTGAAGTTGTGGAGCCAGTTACTATCCCTAGTCAACAACCCCCGTCAAAGCCGACAACTG

The *A. thaliana* gene *AT1G13730* lost an 84-bp-long intron and a 15-bp-long segment from an upstream exon. This deletion did not cause any frame shift events. The active expression of the gene is supported by SRR360152.21059380, SRR360152.24741759, SRR360152.22726069, SRR360152.30624108, SRR360152.18367924, SRR360152.32397916, SRR360152.23981125, SRR360152.10297991, SRR360152.25042258, and SRR360152.11133801 (>10 RNA-Seq reads, others are not shown)

The successful splicing of the target region of *A. lyrata* is supported by SRR800645.3610853, SRR800645.1693978, SRR800645.4088966, SRR800645.3308328, and SRR800644.6239349.

The successful splicing of the target region of *C. rubella* is supported by SRR797557.24557359, SRR797557.21836012, SRR797558.22524872, SRR797558.33296414, SRR797555.15866424, SRR797555.11258853, SRR797557.26748028, SRR797557.16182986, SRR797558.19776321, and SRR797558.4315897 (>10 RNA-Seq reads, others are not shown).

The assembly of the variation site in *A. thaliana* was supported by WGS reads. First, we found >10 WGS reads of *A. thaliana* crossing the deletion site in *A. thaliana*: SRR960120.120339, SRR960120.14454005, SRR960120.15863739, SRR960120.20567266, SRR960120.26277489, SRR960120.18880216, SRR960120.16004304, SRR960120.6101223, SRR960120.14454005 and SRR960120.20973982, others are not shown. In addition, we found >10 *A. thaliana* WGS reads that matched the regions flanking the target variation site (1 kb at each side) in *A. lyrata* (SRR960120.28048868, SRR960120.23139135, SRR960120.16871252, SRR960120.2162581, SRR960120.210217, SRR960120.1131560, SRR960120.18006765, SRR960120.16004304, SRR960120.14454005 and SRR960120.15863739, others are not shown) but no WGS reads of *A. thaliana* that matched the region of *A. lyrata* that are corresponding to the deletion of *A. thaliana* in position, which confirmed the deletion in *A. thaliana.*

# ***AT1G17680***

*A. thaliana* GAACATCCAGAGCTCATCACCAATTTGGCTGATGAGCTAACAAACATTGGGAATTTCCACTCTGCTCTCAAATACTATATAGAAGCAATC

*A. lyrata*  GAACATCCAGAGCTCATTACCAATTTGGCTGATGAGCTAACAAACATTGGGAATTTCCACTCTGCTCTCAAATACTATTTAGAAGTAATC

*C. rubella*  GAACATCCAGAGCTCATCACCGATTTGGCTGATGAGCTAACAAACATTGGGAAATTTCACTCTGCTCTCAAATACTATTTAGAAGCAATC

*A. thaliana* AGTGAACCTGTG------------------------------------------------------------------------------

*A. lyrata*  AGTGAACCTGTGAATgtaaggagatcaccagttctgctctattgtttcctctcttagaagttttgcttcaccctttggataactagtatt

*C. rubella*  AGTGAATCTGTGAATgtaaggcaatcaccaattctgctccattgtttcctctcttccaacttttgcttcaccctttggataactcgtatt

*A. thaliana* ----------------AATGGCAATTTGTTTGTAAAAATTGCTCGCTGTTATATGTCATTGGAAGAAAGAAAACAGGCCATTGTTTTCTA

*A. lyrata*  gtggttttccttgtagAATGGCTATTTGTTTGTGAAAATTGCTCGCTGTTATATGTCATTGGCAGAACGAGAACAGGCCATTGTTTTCTA

*C. rubella*  gtggttttccttgtagAATGGCTATTTATTTGTGAAAATTGCTCGTTCTTATATGTCATTGGCAGAACGTGAACAGGCCATTGTTTTCTA

The *A. thaliana* gene *AT1G17680* has lost a 91-bp-long intron and a 3-bp-long segment from an upstream exon. This deletion did not cause any frame shift events. The active expression of the gene is supported by SRR360152.28684375, SRR360152.21314686, SRR360152.6091237, SRR360152.12680511, SRR360152.288433, SRR360152.362707, SRR360152.12512384, SRR360152.30933920, SRR360152.21084201, and SRR360152.23264313 (>10 RNA-Seq reads, others are not shown).

The successful splicing of the target region of *A. lyrata* is supported by SRR800644.3894610, SRR800644.3935613, SRR800644.2850950, and SRR800645.4296505.

The successful splicing of the target region of *C. rubella* is supported by SRR797556.4048736, SRR797556.15043191, SRR797555.22012371, SRR797558.35196966, SRR797556.17217683, SRR797555.8708096, SRR797558.16975326, SRR797558.7451180, SRR797555.41655160, and SRR797557.16012730 (>10 RNA-Seq reads, others are not shown).

The assembly of the variation site in *A. thaliana* was supported by WGS reads. First, we found six WGS reads of *A. thaliana* crossing the deletion site in *A. thaliana*: SRR960120.13500791, SRR960120.20641358, SRR960120.29073749, SRR960120.28783735, SRR960120.1460997 and SRR960120.9774621. In addition, we found >10 *A. thaliana* WGS reads that matched the regions flanking the target variation site (1 kb at each side) in *A. lyrata* (SRR960120.29315115, SRR960120.29392350, SRR960120.3760969, SRR960120.25255537,SRR960120.23638192, SRR960120.28783735, SRR960120.1460997, SRR960120.14893191,SRR960120.9506 and SRR960120.22989895, others are not shown) but no WGS reads of *A. thaliana* that matched the region of *A. lyrata* that are corresponding to the deletion of *A. thaliana* in position, which confirmed the deletion in *A. thaliana.*

# AT1G48175

*A. thaliana* GTATCAAGGAAGTGTATTATGGATGTCCAAATGATAAATTTGGGGGATGTGGTTCCATTTTGTCGCTTCACTTAGG--------------

*A. lyrata*  GTATAAAGGAAGTGTATTATGGATGTCCAAATGATAAATTTGGGGGATGTGGTTCCATTTTGTCGCTTCACTTGGGCAGTTCTCAGTCGT

*C. rubella*  GTATAAAGGAAGTGTATTATGGATGTGCAAATGATAAATTTGGTGGATGTGGTTCGATCTTATCGCTTCACGTAGGCAGTCCTCAGCCGT

*A. thaliana* ----------------------------------------------------------------------------TAGTGAGGAAGCTC

*A. lyrata*  CAGATATCATgtaagttatacgatcacactacaatctttttaaaaatatgccttgttaattgatttctggt-ttgatagTGAGGAATCTC

*C. rubella*  CTGATACTCGgtaggttctacaatcacacaacagtc-ctataaaaaaatgcctcatcaattgaattctgttggttacagGGAAGAAGCTC

*A. thaliana* AAAGAGGAAAAGGATACAAGTGCAGAGGAGGAATAATGGCAGAAGAAGCTGTCTCTCTTTTCAAATGTTTCTATGA

*A. lyrata*  AAAGAGGAAAAGGGTATAAATGCAGAGGAGGAATAATGGCAGAAGAAGCTGTCTCTCTTTTCAAATGTTTCTATGA

*C. rubella*  AGAGAGGCAAAGGGTACAAGTGCAGAGGAGGAATCATGGCAGAAGAAGCTGTCTCTCTTTTCAAATGTTTCTATGA

The *A. thaliana* gene *AT1G48175* lost an intron and a 24-bp-long segment from an upstream exon, and it simultaneously added a 3-bp-long segment to a downstream exon. This deletion did not cause any frame shift events. The active expression of the gene is supported by SRR360152.16239304, SRR360152.29088743, SRR360152.1111216, SRR360152.24859739, SRR360152.6048015, SRR360152.11085730, SRR360152.11765460, SRR360152.16773042, SRR360152.1447739, and SRR360152.22242025 (>10 RNA-Seq reads, others are not shown).

The successful splicing of the target region of *A. lyrata* is supported by SRR800645.2474390, SRR800645.4829699, SRR800645.3472885, SRR800645.2209575, SRR800644.1156498, SRR800644.978671, SRR800645.70372, SRR800644.136669, SRR800645.944644, and SRR800645.2839790.

The successful splicing of the target region of *C. rubella* is supported by SRR797558.35347361, SRR797558.6319723, SRR797558.28102543, SRR797555.3698923, SRR797556.16733043, SRR797556.28902591, SRR797555.43579143, SRR797556.4619978, SRR797556.27133860, and SRR797558.9216640 (>10 RNA-Seq reads, others are not shown).

The assembly of the variation site in *A. thaliana* was supported by WGS reads. First, we found eight WGS reads of *A. thaliana* crossing the deletion site in *A. thaliana*: SRR960120.16452931, SRR960120.9904171, SRR960120.1995885, SRR960120.6905564, SRR960120.14207801, SRR960120.16101580, SRR960120.22857805 and SRR960120.3303864. In addition, we found >10 *A. thaliana* WGS reads that matched the regions flanking the target variation site (1 kb at each side) in *A. lyrata* (SRR960120.15706966, SRR960120.9566267, SRR960120.12550850, SRR960120.5129409, SRR960120.5691838, SRR960120.7334347, SRR960120.8482242, SRR960120.26543087, SRR960120.3759989 and SRR960120.24665959, others are not shown) but no WGS reads of *A. thaliana* that matched the region of *A. lyrata* that are corresponding to the deletion of *A. thaliana* in position, which confirmed the deletion in *A. thaliana.*

# AT1G54680

*A. thaliana* CTTATGAAAGATGATGTCGACGGCCAAATGAA----------------------------------------------------------

*A. lyrata*  CTAATGAAAGATGATAGGGATAGCCGGATGAATCTATCGgtatgttatatacttgtatat--tagcatctctattgtctatcttctttac

*C. rubella*  GTTATGAAAGATGATATGGACGGTCGAATGAATCTATCGgtatgttatatacttgtatgttataatacctctgttgtgtatcttcttaac

*A. thaliana* ---------------------------------CCAAGGCAATATCTCATCCAAGgtaagatttcctca-aatggacctgtaatgagtgt

*A. lyrata*  tcaatacttttttttgtgtacaatgtagGATACTCAAGGCAATATCTCTTCCAAGgtacgattttctca-aacttacattcaatgaatga

*C. rubella*  ttgatacattttttcttctacaatgtagGGTTCTCAAGGCAATATCTCATCTAAGgtaagatttcttcacattgtatctgtaatgagtgg

The *A. thaliana* gene *AT1G54680* lost a 77-bp-long intron, a 7-bp-long segment from an upstream exon and a 5-bp-long segment from a downstream exon. This deletion did not cause any frameshift events. The active expression of the gene is supported by SRR360152.26765681, SRR360152.32149752, SRR360152.304236, SRR360152.19701743, SRR360152.7988383, SRR360152.22367534, SRR360152.32151824, SRR360152.16875431, SRR360152.11260634, and SRR360152.3777502 (>10 RNA-Seq reads, others are not shown).

The successful splicing of the target region of *A. lyrata* is supported by SRR800645.4363190, SRR800644.4539926, SRR800644.76309, SRR800645.4452478, SRR800644.4532608, SRR800644.3241525, SRR800644.5140739, SRR800645.607320, SRR800645.1353126, and SRR800644.495262 (>10 RNA-Seq reads, others are not shown).

The successful splicing of the target region of *C. rubella* is supported by SRR797558.30488329, SRR797556.10425391, SRR797556.3309409, SRR797556.31725516, SRR797558.16630499, SRR797555.31632544, SRR797558.21017927, SRR797556.10999250, SRR797558.8141341, and SRR797556.21268919... (>10 RNA-Seq reads, others are not shown).

The assembly of the variation site in *A. thaliana* was supported by WGS reads. First, we found >10 WGS reads of *A. thaliana* crossing the deletion site in *A. thaliana*: SRR960120.26375615, SRR960120.6607344, SRR960120.28506342, SRR960120.15689366, SRR960120.17412841, SRR960120.24754657, SRR960120.2199110, SRR960120.7908303,SRR960120.6426964 and SRR960120.7062029, others are not shown. In addition, we found >10 *A. thaliana* WGS reads that matched the regions flanking the target variation site (1 kb at each side) in *A. lyrata* (SRR960120.5371020, SRR960120.9411788, SRR960120.14993656, SRR960120.27664305,SRR960120.20848920, SRR960120.2706780, SRR960120.8940755, SRR960120.12265394,SRR960120.12686160 and SRR960120.18914847, others are not shown) but no WGS reads of *A. thaliana* that matched the region of *A. lyrata* that are corresponding to the deletion of *A. thaliana* in position, which confirmed the deletion in *A. thaliana.*

# AT2G39240

*A. thaliana* TCGGAGATTGAGTGCGATGATAATCTCCAAAATGACTCCAGTCTAGCAATGTTTGATATGGAACTTGACGTTGAAGGCAC----------

*A. lyrata*  TTGAAGATTGGGTGGGATGATATACTCCAAGATGACTCTACCATAGCCATGTTTGATATGGAACTTGACGTTGAAGGCACTACCAATGAA

*C. rubella*  TTGGAGATTGGGTGGGATGATATTCTTCAAGATGACTCCAGTATAGCCATGTTTGATATGGAACTTGACGTTGAAGGCACTATGAATGAA

*A. thaliana* ------------------------------------------------------------------------------------------

*A. lyrata*  GGAGAGGAGgtagggttagtgtg-tttatctgctgtttatgctggatccataaacttggttgaaagtttcaggattacaaaatgtttttt

*C. rubella*  GGAGAGGAGgtagggttactgtgttttatctgctgttcatactgaattcacaaaattggatgaaagtttctgatttacaagatgcttttt

*A. thaliana* ------------------------------------------------------------------------------------------

*A. lyrata*  tctcttatatactatgaaaatttggagagctatctgccatcattcataacatttgattttccttttgcagTTTTCAGTTCGGTCTCTAAA

*C. rubella*  ctct-----tactacgaaaatttagatacatatctgccatcactcatga-atttgatttttcttttgcagTTTCCAGTGCGGTCTCTGAA

*A. thaliana* ------------TCTAGTTTCTAAATTGTTGGACGATTTAATGGTCGAATCTTTTAATCATCTTAAATCTTGTCAAGATGCTGGTCGTCT

*A. lyrata*  TCAAAATGGAAATGTAGTCTCTAAATCGTTGGACGATTTGATGGTCCTATCTTTTCATCATCTTGAATTCTGCCAAGATGCTGGTCGTCT

*C. rubella*  TAAAAATGGAAATGTAGTCTCTGAATTGTTTGACGACTTAATGGTTCTATCTTTTAAGCATCTTGAATCCTGTCAAGATGCTGGTCGTCT

The *A. thaliana* gene *AT2G39240* lost a 150 bp intron, a 19 bp segment from an upstream exon and a 32 bp segment from a downstream exon. This deletion did not cause any frame shift events. The active expression of the gene is supported by SRR360152.3127315, SRR360152.16093704, SRR360152.12597138, SRR360152.1668265, SRR360152.16093704, SRR360152.1668265, SRR360152.3127315, SRR360152.27426238, SRR360152.12597138, and SRR360152.21922573 (>10 RNA-Seq reads, others are not shown).

The successful splicing of the target region of *C. rubella* is supported by SRR797556.8145901, SRR797556.1752627, SRR797557.22475322, SRR797556.8145901, SRR797557.8998330, SRR797555.25884253, SRR797556.25751338, and SRR797556.11817452.

The assembly of the variation site in *A. thaliana* was supported by WGS reads. First, we found nine WGS reads of *A. thaliana* crossing the deletion site in *A. thaliana*: SRR960120.23882047, SRR960120.5537616, SRR960120.12785545, SRR960120.13873232, SRR960120.928725, SRR960120.24490241, SRR960120.3350980, SRR960120.15668342 and SRR960120.29430689. In addition, we found >10 *A. thaliana* WGS reads that matched the regions flanking the target variation site (1 kb at each side) in *A. lyrata* (SRR960120.29401071, SRR960120.18542030, SRR960120.61033, SRR960120.5929404, SRR960120.11074575, SRR960120.12695338, SRR960120.23185232, SRR960120.22889260, SRR960120.402909 and SRR960120.20131211, others are not shown) but no WGS reads of *A. thaliana* that matched the region of *A. lyrata* that are corresponding to the deletion of *A. thaliana* in position, which confirmed the deletion in *A. thaliana.*

# AT2G47910

*A. thaliana* CCTCCAACGACCGGCCGGTACGAAGTAGTGATCGACAACGACTCAATTGGGCGGCTTGACCTATCCCCGTTTCAGAGAGCCATCGGCATA

*A. lyrata*  CCTCCGACGACGGGACGGTACGAAGTAGTGATCGACAACGACTCAATTGGGCGGCTTGACCTATCCCCGTTTCAGAGAGCCACCGGCATA

*C. rubella*  CCTCCCAAGACCGGGCGCTACGAGGTTGTGATCGACAACGACTCCATTGCGCGGCTTGACCTGTCCCCGTTTCAGACAGCCACCGGCATA

*A. thaliana* ACCTCGCCT-----------------------------------------------------GATGATTTTG-GTGTAGCTGAACCAAAA

*A. lyrata*  AGCTCACCTTCCTCAGgtaggtatacatatttatttatccctcacgtgaatgtgattgattcgttgactgtgtgtgtagCTGAACCAAAA

*C. rubella*  ACATCACCTTCTCTAGgtaggtatacattataatttagtattgatgtcacggcagtttgtttgtttgtttggtgtatagCTGAACCAAAA

*A. thaliana* CGGTATTTGGATCGAACCATCGGATTCACCATCAATTACAAGAGAGAAGACCCCGGGGATCCGCGGGAGCTGTCAGAGTACCCGGACGTA

*A. lyrata*  CGGTATTTGGATCGAACCATCGGATTCACCATCAAGTACAGGAGAGAAGATCCCGGGGATCCGCGGGAGCTGTCAGAGTACCCAGACATA

*C. rubella*  GAGTATTTGGATCGAAGCATCGGATTCACGATCAAGTACAAAAGAGAAGACCCCGGGGATCCGCGGGAGCTGTCAGAGTACCCGGACATA

The *A. thaliana* gene *AT2G47910* has lost an intron and a 7 bp segment from an upstream exon, and it simultaneously added a 16-bp-long segment to a downstream exon. This deletion did not cause frame-shifts. The active expression of the gene is supported by SRR360152.24333368, SRR360152.16940375, SRR360152.17374775, SRR360152.2942058, SRR360152.11774793, SRR360152.21499125, SRR360152.26799519, SRR360152.19490680, SRR360152.22272037, and SRR360152.27370716 (>10 RNA-Seq reads, others are not shown).

The successful splicing of the target region of *A. lyrata* is supported by SRR800645.2810522, SRR800645.2430240, SRR800645.668931, and SRR800645.2447641.

The successful splicing of the target region of *C. rubella* is supported by SRR797557.20091099, SRR797558.13921504, SRR797558.19097366, SRR797556.3615337, SRR797558.23865373, SRR797555.42421773, SRR797555.33574132, SRR797556.27862602, SRR797558.38179961, and SRR797556.1142917 (>10 RNA-Seq reads, others are not shown).

The assembly of the variation site in *A. thaliana* was supported by WGS reads. First, we found four WGS reads of *A. thaliana* crossing the deletion site in *A. thaliana*: SRR960120.6306928, SRR960120.13653891, SRR960120.6306928 and SRR960120.15515858, others are not shown. In addition, we found >10 *A. thaliana* WGS reads that matched the regions flanking the target variation site (1 kb at each side) in *A. lyrata* (SRR960120.13134080, SRR960120.16380933, SRR960120.19018814, SRR960120.19550233,SRR960120.28328175, SRR960120.6306928, SRR960120.15515858, SRR960120.22687927,SRR960120.20978940 and SRR960120.25642247, others are not shown) but no WGS reads of *A. thaliana* that matched the region of *A. lyrata* that are corresponding to the deletion of *A. thaliana* in position, which confirmed the deletion in *A. thaliana.*

# AT3G07160

*A. thaliana* TTCGTGGGATGATGTATTATAGGAAAGCCCTCATGCTTCAGTCTTATCTGGAAAGAAAAGCTGG--------------------------

*A. lyrata*  TTCGCGGGATGATGTATTATAGGAAAGCCCTCATGCTTCAGTCTTATCTGGAGAGAAAGGCTGGAAGAGgtaaagaggctaaagaccttt

*C. rubella*  TGCGAGGTATGATGTATTACCGAAGGGCTTTGATGCTTCAGAGTTTCTTAGAGAGACGGGGCTTGGGAGgt-------------acatat

*A. thaliana* ----------------------------------------------------------CAATGACGCTACAGACGCTGAAGGATTTGAGT

*A. lyrata*  ttattatatcattttttggccaaacttttacttagggctatctcactattacagACGATGAGGACGCAACAGACGCTGAAGGCTTTGAGT

*C. rubella*  ttcttttcttttttttgcagcattcccttataaggaaaggcatatctgtaagagcagttttttccgttacagTGGATGACGGGTTTGAAT

*A. thaliana* TATCTCCAGAAGCAAGGGCCCAAGCAGATCTCAAGTTCACATATGTTGTCACATGCCAGATATATGGAAGACAAAAAGAAGATCAAAAAC

*A. lyrata*  TATCTCCAGAAGCAAGGGCCCAGGCAGATCTGAAGTTTACATATGTCGTCACATGCCAGATATATGGAAGACAGAAAGAAGATCAAAAAC

*C. rubella*  CATCACCTGAAGCACGAGCTCAAGCAGACTTGAAATTTACGTATGTTGTGTCATGCCAAATATATGGTCAACAGAAACAGCAAAAGAAAC

The *A. thaliana* gene *AT3G07160* lost a 75-bp-long intron, a 5-bp-long segment from an upstream exon and a 4-bp-long segment from a downstream exon. This deletion did not cause any frame shift events. The active expression of the gene is supported by SRR360152.15086175, SRR360152.22815101, SRR360152.31322607, SRR360152.12436459, SRR360152.19693156, SRR360152.27335260, SRR360152.25420334, SRR360152.7586576, SRR360152.2707219, and SRR360152.11441003 (>10 RNA-Seq reads, others are not shown).

The successful splicing of the target region of *A. lyrata* is supported by SRR800644.4892285, SRR800644.1373015, SRR800644.3096312, SRR800644.739606, SRR800644.2407157, SRR800645.57224, SRR800644.564548, SRR800645.4416716, SRR800644.3398842, and SRR800645.1713467 (>10 RNA-Seq reads, others are not shown).

The successful splicing of the target region of *C. rubella* is supported by SRR797555.1169211, SRR797555.4056183, SRR797558.24697973, SRR797558.10304319, SRR797555.33279593, SRR797558.36730613, SRR797557.29452042, SRR797556.14144849, SRR797556.21587256, and SRR797558.25932746... (>10 RNA-Seq reads, others are not shown)

The assembly of the variation site in *A. thaliana* was supported by WGS reads. First, we found >10 WGS reads of *A. thaliana* crossing the deletion site in *A. thaliana*: SRR960120.12119367, SRR960120.6293033, SRR960120.28818498, SRR960120.16266861, SRR960120.13930923, SRR960120.19277573, SRR960120.10628817, SRR960120.13402812,SRR960120.17663584 and SRR960120.20297169, others are not shown. In addition, we found >10 *A. thaliana* WGS reads that matched the regions flanking the target variation site (1 kb at each side) in *A. lyrata* (SRR960120.13297569, SRR960120.24241610, SRR960120.14883702, SRR960120.20045920,SRR960120.8936956, SRR960120.10628817, SRR960120.17663584, SRR960120.4944196,SRR960120.16042697 and SRR960120.13172834, others are not shown) but no WGS reads of *A. thaliana* that matched the region of *A. lyrata* that are corresponding to the deletion of *A. thaliana* in position, which confirmed the deletion in *A. thaliana.*

# AT3G16940

*A. thaliana* GCAGCTACAACATCGGGGGACTCAATCTCTAGTCCAATCTCCGTCTCGGAACAAACATTCCCTAATCGCGTGGCAGCTGAAGATATT---

*A. lyrata*  GCAGGTACAACATCGGGGGACTCAAACTCTAGTCCAATCTCCGTCTCTGATCAAGCATTCCCAAATCTCGTGACAGCTGAAGATATTGAT

*C. rubella*  GCAGCTACTTCATCGGGAGAGTCAAACTCTAGTCCAATAGAGGTCTCCGACCAGACATTCCCAAATCTCATGACAGCTGAACATATTGAT

*A. thaliana* ------------------------------------------------------------------------------------------

*A. lyrata*  TTTAGCATTGAGAATTCACGTTATCTAGgtgagaagcaatctttaagtgatctcggtttttcacagtttttatttgctgtttgcaaactt

*C. rubella*  TTTAGCATTGAGAACTCACGTTATATAGgtgagaacaatcttttacgtgatatcggtttttcacagtttttatttgctgtttgcaaacta

*A. thaliana* -------------------------------------------------------------------------GATACAGTTGTTAGAAA

*A. lyrata*  taaatgctttctctgatttcttcatttggacacttatcacactttcttttggtggctcacagCAAGCAACAACGATACCGTTGTTAGAAA

*C. rubella*  taaatgtgttctctggtctcttcatatggatacttatcacactatcttttggtggctcacagCAAGCAACAACAGTCCAGTTGTTAGAAA

*A. thaliana* TCATGATATTAGCCTTCATGATATCAATACGCTTGATTGGGATGAGCTGCTAGTACCAACCGATCTTAATAACCAATCTGCACCAACCGT

*A. lyrata*  TCATGATATTAGCCTTCATGATATCAATACGCTTGATTGGGATGAGCTGCTAGTACCAACCGATCTTAATAACCAATCTGCACCAACCGT

*C. rubella*  TCATGAAATTAGCCTTCACGATATCAATACACTCGATTGGGATGAGCTGCTAATACCAACCGATCTTCACAACCAATCTGCACCAACCGA

The *A. thaliana* gene *AT3G16940* lost a 124-bp-long bp intron, a 31-bp-long segment from an upstream exon and an 11-bp-long segment from a downstream exon. This deletion did not cause any frame shift events. The active expression of the gene is supported by SRR360152.2510744, SRR360152.2917215, SRR360152.4043845, SRR360152.14039505, SRR360152.19270223, SRR360152.30856474, SRR360152.8757128, SRR360152.8971853, SRR360152.22011008, and SRR360152.6288855 (>10 RNA-Seq reads, others are not shown).

The successful splicing of the target region of *A. lyrata* is supported by SRR800645.1537779, SRR800645.1504887, SRR800644.4036599, SRR800644.5868694, SRR800645.1835200, SRR800644.1170058, and SRR800644.5428197.

The successful splicing of the target region of *C. rubella* is supported by SRR797555.37815922, SRR797556.786899, SRR797556.30873260, SRR797558.24314421, SRR797555.24651767, SRR797558.6549053, SRR797556.30873260, SRR797558.21290519, SRR797555.15794414, and SRR797556.18181146 (>10 RNA-Seq reads, others are not shown).

The assembly of the variation site in *A. thaliana* was supported by WGS reads. First, we found >10 WGS reads of *A. thaliana* crossing the deletion site in *A. thaliana*: SRR960120.22688574, SRR960120.28262029, SRR960120.28947085, SRR960120.9218860, SRR960120.5738814, SRR960120.5482744, SRR960120.17883636, SRR960120.25459943, SRR960120.8086381 and SRR960120.28947085, others are not shown. In addition, we found >10 *A. thaliana* WGS reads that matched the regions flanking the target variation site (1 kb at each side) in *A. lyrata* (SRR960120.7994083, SRR960120.17934461, SRR960120.29235651, SRR960120.26879634, SRR960120.22143245, SRR960120.9218860, SRR960120.25459943, SRR960120.28947085, SRR960120.16599283 and SRR960120.3243680, others are not shown) but no WGS reads of *A. thaliana* that matched the region of *A. lyrata* that are corresponding to the deletion of *A. thaliana* in position, which confirmed the deletion in *A. thaliana.*

# AT3G17330

*A. thaliana* gttgataggctggttcatgaaatttacttatcactcttgcgttgcaaattgcctaaaattctggaaccttttttcagGTTATGATTC---

*A. lyrata*  gttgata-gctggctcactaactttacttgttactcttgcgttgcaaattccctaagttactgg-attttttttcagGTTATGATTCATC

*C. rubella*  gttggta-gctgcttcacgaaatttacttatctctcttgcgctgccaattccttaatgtactggatttctttttcagGTTATGATTCATC

*A. thaliana* ------------------------------------------------------------------------------------------

*A. lyrata*  TGTCGAATGGGAGAACCGTCAGGTTATTCTTGGTGTAGATGGTTCAGAGGTCCAATACACGgtgtgtgaaacttgctgtttttttttttt

*C. rubella*  CATCGATTGGGAGAACTGTCAGG------TTGGTGTAGATGGTTCAGAGGTCCAATACATGgtgcgtgaacctctcacttgctgtttttt

*A. thaliana* ------------------------------ATCTGTGGGTCTTCAGGGTGGGCAAAATGAAAATGCTCCCTATATCTGCTATACACCAAG

*A. lyrata*  tttgttattgtcctatctgctaacattctgatctgtgcgtcttcagGGTGGGCAAAATGAAAATTCTCCTTATATCTGCTATACACCAAG

*C. rubella*  tttgttatcttcttattggctaaaattctaatct-tgcatcttcagGGTGGGCAGAATGAAAATTCTCCCTATATATGCTATACACCAAG

*A. thaliana* TTATGGATATGCACAGTCTCCCTACAATCCCTACAATCCTTACATACCAGGAGCTTCAATAGGCGTTGATTCTTCTTTTGTTGGATTCCA

*A. lyrata*  TTATGGATATGCACAGTCTCCCTACAATCCCTACAATCCTTACATACCAGGCGCATCAATAGGCGTTGACTCTTCTTTTCTTGGATTCCA

*C. rubella*  TTATGGATATGCGCAGTCTCCTTACAATCCGTACAATCCTTACATACCAGGCGCGTCAATAGGTGTTGACTCTTCGTTTGTTGGATTCCA

The *A. thaliana* gene *AT3G17330* lost an intron and a 64-bp-long segment from an upstream exon, and it simultaneously added a 16-bp-long bp segment to a downstream exon. This deletion did not cause any frame shift events. The active expression of the gene is supported by SRR360152.20833245, SRR360152.24357688, SRR360152.10209078, SRR360152.17089178, SRR360152.293240, SRR360152.20654058, SRR360152.22717165, SRR360152.13063505, SRR360152.23141469, and SRR360152.15675719 (>10 RNA-Seq reads, others are not shown).

The successful splicing of the target region of *A. lyrata* is supported by SRR800644.6623417.

The successful splicing of the target region of *C. rubella* is supported by SRR797558.27633098, SRR797555.6432906, SRR797555.27367357, SRR797558.34234888, SRR797557.30469237, SRR797557.25922451, SRR797558.30752656, SRR797558.26471656, SRR797558.10598217, and SRR797555.16688930 (>10 RNA-Seq reads, others are not shown).

The assembly of the variation site in *A. thaliana* was supported by WGS reads. First, we found six WGS reads of *A. thaliana* crossing the deletion site in *A. thaliana*: SRR960120.24705543, SRR960120.18608374, SRR960120.7736209, SRR960120.28449230, SRR960120.22586641 and SRR960120.6576001. In addition, we found >10 *A. thaliana* WGS reads that matched the regions flanking the target variation site (1 kb at each side) in *A. lyrata* (SRR960120.18792407, SRR960120.13352814, SRR960120.10438798, SRR960120.28480064, SRR960120.22280717, SRR960120.2957272, SRR960120.18919131, SRR960120.17847233, SRR960120.8578318 and SRR960120.18039091, others are not shown) but no WGS reads of *A. thaliana* that matched the region of *A. lyrata* that are corresponding to the deletion of *A. thaliana* in position, which confirmed the deletion in *A. thaliana.*

# AT3G27670

*A. thaliana* TCATCAATGGCTTCCCTTTGTTGCTCATCCCGCCACGAAGCACTACCGATATTCAGATTGCTTATGCGATGTCTAAAATATATCCCGGGA

*A. lyrata*  TCATCAATGGCTTCCCTTTGTTGTTCATCCCGCAAAGAAGCACTTCCGATATTCAGGTTGCTATTGCGTTGCCTAAAATATATCCCGGGA

*C. rubella*  TCATCGATGGCTTCTCTTTGTTGTTCATCCCGCCACGAAGCACTGCCGATATTCAGATTGCTTATGCGTTGCTTAAAATATATCCCGGGG

*A. thaliana* AATAATCTGGAAGTA---------------------------------------------------------------------------

*A. lyrata*  AAAAATCTGGAAgtaagtttacatgattagcagtgattttggtgaatgcaagtaatgggatgcaatattagtatacagttctaaatttct

*C. rubella*  AAAAACCTCGAAgtaagtatacatgattagcagtcgctttggtgaatgaaaataatgg--tgcaatattaggattcagttctaaatttct

*A. thaliana* ---------------------------------ATTGTCAAGATCCTAGTGGATGCCTACACTGTGGTTGTGAGAGACTTGGTTGGAACC

*A. lyrata*  gaaactgttcagGACAATAGAAATTTCAGCTGCATTGTCAAGACTCTAGTGGATGCATACACTGTGGTTGTGAGAGACTTGGTTGGAACT

*C. rubella*  gaaactgttcagGACAATAGAAATTTCAGCTGTATTGTCAAGACTCTAGTGGATGCTTATACTGTGGTTGTGAGAGACTTGGTTGGAACT

The *A. thaliana* gene *AT3G27670* lost an intron and a 21-bp-long segment from a downstream exon, and it simultaneously added a 3-bp-long segment to an upstream exon. This deletion did not cause any frame shift events. The active expression of the gene is supported by SRR360152.4004907, SRR360152.28886274, SRR360152.5505289, SRR360152.14246481, SRR360152.20524593, SRR360152.5028830, SRR360152.6311851, SRR360152.20910486, SRR360152.14644407, and SRR360152.27510931 (>10 RNA-Seq reads, others are not shown).

The successful splicing of the target region of *C. rubella* is supported by SRR797555.18899896, SRR797556.20579178, SRR797555.42723642, SRR797558.36721286, SRR797555.42247597, SRR797557.4500039, SRR797555.37246380, SRR797558.40506390, SRR797555.16678492, and SRR797558.35764285 (>10 RNA-Seq reads, others are not shown).

The assembly of the variation site in *A. thaliana* was supported by WGS reads. First, we found >10 WGS reads of *A. thaliana* crossing the deletion site in *A. thaliana*: SRR960120.11894558, SRR960120.7151304, SRR960120.2300009, SRR960120.15946896, SRR960120.27714778, SRR960120.2361907, SRR960120.28818787, SRR960120.12056062, SRR960120.2807632 and SRR960120.4200652, others are not shown. In addition, we found >10 *A. thaliana* WGS reads that matched the regions flanking the target variation site (1 kb at each side) in *A. lyrata* (SRR960120.20682886, SRR960120.1692725, SRR960120.13933214, SRR960120.18143351, SRR960120.20138642, SRR960120.28818787, SRR960120.12056062, SRR960120.2807632, SRR960120.4200652 and SRR960120.22651650, others are not shown) but no WGS reads of *A. thaliana* that matched the region of *A. lyrata* that are corresponding to the deletion of *A. thaliana* in position, which confirmed the deletion in *A. thaliana.*

# AT3G54170

*A. thaliana* ttaattcatggtttttgtttaaggttggattggacattaatttctcatctgtatcattcagGTAATAGAAGAAGCTTCGGTGATCTTGAA

*A. lyrata*  caaattcatggtttttggtt-agattggattggaatttcacttttcatctgtatcattcagGTAATAAAAGAAGCTTCGGTGATCTTGAA

*C. rubella*  acaatttatggtttcgtggttagattggagtgaagtttctttagttatctgtatcattcagGTAATAAAAGAAGCTTCGGTGATCTTGAA

*A. thaliana* GACGATGAAGATGATATTTTCGGATCAACAACGGTAGCG---------------------------------------------------

*A. lyrata*  GACGATGAAGATGATATTTTCGGATCAAAAAAGgtagcgcctttgaattttatatatcacaagttcagtttttactcacttgcttgatca

*C. rubella*  GACGATGAAGATGATATTTTCGGATCAAAAAAGgtaggccttttcgattttttatatcgcaagttcagtttttgctcacctgcttgatca

*A. thaliana* ------------------------------------------CCTGGTGTTAGAACTGGGATGATTTTGTCTCTCCGAGGAAGgttatt-

*A. lyrata*  atggtcgccgtttagGGTCGAACTAAAGTGGAGGAAGCTGCACCTGGTGTTGCAACTGGGATGATTTTGTCACTTCGTGAGAGgtttttt

*C. rubella*  atgatcgttgtttagGGTCGAGCTAAAGTGGAGGAAGCTGCACCCGGTGTTGCCACTGGGATGATTTTGTCACTCCGAGAGAGgtttttt

The *A. thaliana* gene *AT3G54170* lost an intron and a 27-bp-long segment from a downstream exon, and it simultaneously added a 6-bp-long segment to an upstream exon. This deletion did not cause any frame shift events. The active expression of the gene is supported by SRR360152.11323788, SRR360152.1778868, SRR360152.5834863, SRR360152.5938194, SRR360152.14734831, SRR360152.23862652, SRR360152.27012416, SRR360152.15611327, SRR360152.30027776, and SRR360152.6686698 (>10 RNA-Seq reads, others are not shown).

The successful splicing of the target region of *A. lyrata* is supported by SRR800645.4677626, SRR800644.92771, SRR800645.3532813, SRR800644.5179699, SRR800644.4321490, SRR800644.1399233, SRR800645.868460, SRR800645.696381, SRR800645.4163402, and SRR800644.3992202.

The successful splicing of the target region of *C. rubella* is supported by SRR797555.44635399, SRR797556.7667091, SRR797558.27392251, SRR797555.8884184, SRR797557.11892268, SRR797556.1668287, SRR797555.28847333, SRR797557.30882684, SRR797558.13221308, and SRR797557.17643110 (>10 RNA-Seq reads, others are not shown).

The assembly of the variation site in *A. thaliana* was supported by WGS reads. First, we found >10 WGS reads of *A. thaliana* crossing the deletion site in *A. thaliana*: SRR960120.1241187, SRR960120.27331935, SRR960120.17439162, SRR960120.16230507, SRR960120.1267922, SRR960120.11373780, SRR960120.24393806, SRR960120.7113862, SRR960120.27331935 and SRR960120.14014546, others are not shown. In addition, we found >10 *A. thaliana* WGS reads that matched the regions flanking the target variation site (1 kb at each side) in *A. lyrata* (SRR960120.22349341, SRR960120.12404344, SRR960120.14302444, SRR960120.18392075, SRR960120.2840380, SRR960120.27331935, SRR960120.16712888, SRR960120.14138693, SRR960120.1729467 and SRR960120.4093984, others are not shown) but no WGS reads of *A. thaliana* that matched the region of *A. lyrata* that are corresponding to the deletion of *A. thaliana* in position, which confirmed the deletion in *A. thaliana.*

# AT3G63400

*A. thaliana* ATATTCATCCTCCTATGAGTCTAGTTCTTCCAGTGATGGGAAGCATAGGAAGAGGAAAACAACAAGACACAAAGGCCGACGCGGGGAAAG

*A. lyrata*  ATATTCATCCTCCTATGAGTCTAGTTCTTCAAGTGATGGGAAGCATAGGAAGAGGAAAACAAAGAGACACAAAGGCGGACGCGGGGAAAG

*C. rubella*  ATATTCATCCTCCTATGAGTCTAGTTCTTCCAGTGATGGAAAGCGTAGGAAGAGGAGAACAAAGAGACACAAAGGCCGACGCGGGGAACA

*A. thaliana* AAAGAGTAAAGGACGAAGCGGGAAAAAGAAAGCTAGAAAACC------------------------------------------------

*A. lyrata*  AAAGATTAAAGGACGAAACGGGAAAAAGAAAGCTAGACAACCTCGGCGCAGgtttgacaattagttcattggtaattagtttattcgctg

*C. rubella*  AAAAATTAAAGGACGAAATGGCAAAAAGAAAGCTAGAAAACCTCGgcgcaggtttcactatttgttcatggtaatttaatttcttctctg

*A. thaliana* ---------------------------------------AAGCACGAATAGTTCAAGTGACACGGAGAGTAGCAGCAGTTCTGATGATGA

*A. lyrata*  ccagttgttttacttgattttattagtggttccttacagAAGCACGTATAGTTCAAGTGACACAGAGAGTAGCAGCAGTTCTGATGATGA

*C. rubella*  ccaattgttttgcttacttttattagtggttcctttcagAAACCCGGATAGTTCAAGTGACAGCGAGAGTAGCAGCAGTTCTGACGATGA

The *A. thaliana* gene *AT3G63400* lost a 78-bp-long intron and a 9 bp segment from a upstream exon. This deletion did not cause any frame shift events. The active expression of the gene is supported by SRR360152.27403450, SRR360152.12859074, SRR360152.24508794, SRR360152.10309725, SRR360152.32188377, SRR360152.17035349, SRR360152.22804144, SRR360152.26261618, SRR360152.27207222, and SRR360152.10142430.

The successful splicing of the target region of *A. lyrata* is supported by SRR800644.1526818, SRR800645.900671, SRR800644.5202877, SRR800644.2214742, SRR800644.6267158, SRR800644.4694443, SRR800645.3753130, SRR800645.1219398, SRR800645.1379099, and SRR800645.3097791 (>10 RNA-Seq reads, others are not shown).

The successful splicing of the target region of *C. rubella* is supported by SRR797557.13753730, SRR797557.1541013, SRR797556.22083392, SRR797558.31651270, SRR797555.17879029, SRR797558.33542365, SRR797558.15047313, SRR797555.37357797, SRR797558.17869673, and SRR797558.11527221 (>10 RNA-Seq reads, others are not shown).

The assembly of the variation site in *A. thaliana* was supported by WGS reads. First, we found >10 WGS reads of *A. thaliana* crossing the deletion site in *A. thaliana*: SRR960120.8630403, SRR960120.4887982, SRR960120.2121112, SRR960120.21695879, SRR960120.26843659, SRR960120.11703170, SRR960120.23261136, SRR960120.11268279, SRR960120.18535523 and SRR960120.16534194, others are not shown. In addition, we found >10 *A. thaliana* WGS reads that matched the regions flanking the target variation site (1 kb at each side) in *A. lyrata* (SRR960120.7954073, SRR960120.12373531, SRR960120.26027081, SRR960120.13723439, SRR960120.19383382, SRR960120.24782703, SRR960120.2121112, SRR960120.5627223, SRR960120.20096828 and SRR960120.22852158, others are not shown) but no WGS reads of *A. thaliana* that matched the region of *A. lyrata* that are corresponding to the deletion of *A. thaliana* in position, which confirmed the deletion in *A. thaliana.*

# AT4G23040

*A. thaliana* GAGTATCGTGGGAACCTCAATAGAGCTGTGAATGCATACTTTACTCATGGAGATCAAAATTCAT--------------------------

*A. lyrata*  GAGTATCGTGGGAATCTCAATAGAGCTGTGAATGCATACTTTACTCATGGAGATCAAAATTTgtatgccctcctcccactgtttattttg

*C. rubella*  GAATACCGTGGGAATCTCAATCGAGCTGTGAATGCATACTATAATCATGGAGATCAGAATTCgtatgccctcccactcttattttttgcg

*A. thaliana* ------------------------------------------------------------------------------------------

*A. lyrata*  ttacctattctttgcatgttttttagacatgcatgtgacacgttctcatcatctttatgaagGCTACATGAAGCCCCAGCTAATATCCCT

*C. rubella*  tattatataccttgcatgtttttaacatgctggtgtcacat-tcttaatcatctttgtgcagACTACATGAAACTCCAGCTAATTTTCCT

*A. thaliana* ----ATGATGCAATGGATATAGATGATGGTGTGACTCCAGTCCATCCAAACTTTGGGAGAAGTCTATTTCCATTTGTTAGTCATCCAAGG

*A. lyrata*  CAGGATGATGCAATGGATATAGATGATGGTGTGACTCCAGTCCATCCAAACTTTGGGACAAGTCTATTTCCATTTGTTAGTCATCCAAGG

*C. rubella*  CGGGATGATGCAATGGATATTGATGATGATGTGACTCCAGCCCATCCAAACTTTGAAAGAAGTCCATTTCCAACTGTTAGTCACCCAAGG

The *A. thaliana* gene *AT4G23040* lost an intron and a 32-bp-long segment from the downstream exon, and it simultaneously added a 2-bp-long segment to an upstream exon. This deletion did not cause any frame shift events. The active expression of the gene is supported by SRR360152.3238461, SRR360152.18104594, SRR360152.20454335, SRR360152.27344127, SRR360152.1146969, SRR360152.28121666, SRR360152.14678658, SRR360152.16839416, SRR360152.8826248, and SRR360152.24798670 (>10 RNA-Seq reads, others are not shown).

The successful splicing of the target region of *A. lyrata* is supported by SRR800644.2009396, SRR800645.4685526, SRR800645.4999506, SRR800644.3481881, and SRR800644.4137441.

The successful splicing of the target region of *C. rubella* is supported by reads: SRR797555.35640716, SRR797558.19245186, SRR797555.9072976, SRR797557.17846944, SRR797556.13338670, SRR797556.2093210, SRR797555.42896255, SRR797556.5368078, SRR797557.5765184, and SRR797558.19740692... (>10 RNA-Seq reads, others are not shown).

The assembly of the variation site in *A. thaliana* was supported by WGS reads. First, we found nine WGS reads of *A. thaliana* crossing the deletion site in *A. thaliana*: SRR960120.4172388, SRR960120.21321584, SRR960120.8536266, SRR960120.17881849, SRR960120.18492825, SRR960120.14207228, SRR960120.11217110, SRR960120.24760909 and SRR960120.2698857. In addition, we found >10 *A. thaliana* WGS reads that matched the regions flanking the target variation site (1 kb at each side) in *A. lyrata* (SRR960120.20421362, SRR960120.4088488, SRR960120.25341175, SRR960120.12676844, SRR960120.23390351, SRR960120.21321584, SRR960120.4172388, SRR960120.26543389, SRR960120.9148561 and SRR960120.3649972, others are not shown) but no WGS reads of *A. thaliana* that matched the region of *A. lyrata* that are corresponding to the deletion of *A. thaliana* in position, which confirmed the deletion in *A. thaliana.*

# AT4G38530

*A. thaliana* ACAAGAACTCCAACGGTTAAAGAGACTTCGTGGAACAGAGTAGCG---------------------------------------------

*A. lyrata*  ACAAGAACTCCAATGGTTAAAGAGACTTCGTGGAGCAGAGTAGCGAGTgtaagaataagtgatttaacgcctcctagtgggaacgatttt

*C. rubella*  ACAAGAACTCCAATGGTTAAAGAGACTTCTTGGAGCATACCAGCAAgtgtgagaagaattgatctaa--ccgcgaaatgggaatggtttt

*A. thaliana* ------------------------------------------------------------AACAAGATTCTTGAAGAAAAAGATATGGAG

*A. lyrata*  gcaaacattcataaattcaatctgctgtaacagAGTACTAATATGGCACGGGGAGGAGAAAACAAGATTCTTGAAGAAGGAGAATTGGAG

*C. rubella*  gcaaacattcatatattttctctgttgtggcagagtaataacatggcatggagaagagAAAACAAGATTCTTGAAGAAGAAGAATTGGAG

The *A. thaliana* gene *AT4G38530* lost a 75-bp-long intron, a 3-bp-long segment from an upstream exon and a 27-bp-long segment from a downstream exon. This deletion did not cause any frame shift events. The active expression of the gene is supported by SRR360152.8531672, SRR360152.8818866, SRR360152.1293209, SRR360152.2658615, SRR360152.16866637, SRR360152.15139663, SRR360152.31033583, SRR360152.30367551, SRR360152.13304360, and SRR360152.5402558 (>10 RNA-Seq reads, others are not shown).

The successful splicing of the target region of *A. lyrata* is supported by SRR800645.3376586, SRR800644.903420, SRR800645.2283477, and SRR800644.1229623.

The successful splicing of the target region of *C. rubella* is supported by SRR797556.12031213, SRR797555.14211971, SRR797555.16189005, SRR797558.33333107, SRR797558.15344028, SRR797558.26463556, SRR797555.36825813, SRR797558.1010442, SRR797555.21412474, and SRR797558.38486730 (>10 RNA-Seq reads, others are not shown).

The assembly of the variation site in *A. thaliana* was supported by WGS reads. First, we found eight WGS reads of *A. thaliana* crossing the deletion site in *A. thaliana*: SRR960120.23904272, SRR960120.12902752, SRR960120.14633515, SRR960120.24321754, SRR960120.400392, SRR960120.18229660, SRR960120.13147079 and SRR960120.16880165. In addition, we found >10 *A. thaliana* WGS reads that matched the regions flanking the target variation site (1 kb at each side) in *A. lyrata* (SRR960120.7079008, SRR960120.20623095, SRR960120.27643584, SRR960120.4901306, SRR960120.29137375, SRR960120.17825915, SRR960120.15699371, SRR960120.22878754, SRR960120.27133084 and SRR960120.3017407, others are not shown) but no WGS reads of *A. thaliana* that matched the region of *A. lyrata* that are corresponding to the deletion of *A. thaliana* in position, which confirmed the deletion in *A. thaliana.*

# AT5G24470

*A. thaliana* TGGAGTGCGCAAAGTCTCAGTTTAATGAGACACGGCTTCTAGCAAATGAGTTGCAGAGTAAGCAAGC-----------------------

*A. lyrata*  TGGAGTGCGCAAAGTCTCAGTTCAATGAGACACGGCTTCTAGCAAATGAGATGCAGAgtaagcaagctttttttatttttttttggcaag

*C. rubella*  TGGAGTGCGCAAAGTCTCAGTTTAGGGAGACACGGGTTCTAGTGAATGAGATGCAGAgtaagcgagctttcatctattttgtctagcaag

*A. thaliana* ----------------------------------------------------------------------------AGAAGCCATTGACT

*A. lyrata*  ctatctgactcttaaaaagttcatcagaatcattgattgacctacatgtttttgtaaattctatagATTCATCGAAAAAGGCCATTGACT

*C. rubella*  acatctgactcttcaaaagtttcatcagaatattaattcaccttcatgtttatgtcaactctacagACGCATCGAAAGAAGCCATTGACT

*A. thaliana* TCATGGGAGCATCGTTTAGAAGAACTGGACGACGTAACAGAGAAGAAAGTGTTGCTCAATACGAATCTCGGATAGAGCTTGATCTTTCTC

*A. lyrata*  TGATGGGAGCATCGTTTAGAAGAACTGGACAACGTAACAGAGAAGAAAGTGCTGCCCAATACGAATCTCGGATAGAGCTTGATCTCTCTC

*C. rubella*  TCATGGGAGCATCGTTTAGAAGAACTGGACAACGTAACAAAGAAGAAAGTGTGGCCCAATACGAATCTCGGATAGAGCTTGATCTCTCTC

The *A. thaliana* gene *AT5G24470* lost an intron and a 10-bp-long segment from a downstream exon, and it simultaneously added a 10-bp-long bp segment to an upstream exon. This deletion did not cause any frame shift events. The active expression of the gene is supported by SRR360152.8222316, SRR360152.15672583, SRR360152.2217623, SRR360152.2978998, SRR360152.25100762, SRR360152.17037241, SRR360152.3859880, SRR360152.3951329, SRR360152.20342269, and SRR360152.24619030 (>10 RNA-Seq reads, others are not shown).

The successful splicing of the target region of *A. lyrata* is supported by SRR800645.3911397, SRR800644.1283616, SRR800644.1249712, SRR800644.3010620, SRR800644.3299316, SRR800645.1360284, SRR800644.5286228, SRR800644.6400246, SRR800645.42831, and SRR800644.3574998 (>10 RNA-Seq reads, others are not shown).

The successful splicing of the target region of *C. rubella* is supported by SRR797557.29170417, SRR797555.21329388, SRR797558.36095310, SRR797555.30451227, SRR797558.17411810, SRR797556.13061646, SRR797558.19915336, SRR797558.38883869, SRR797557.20368009, and SRR797556.18858392 (>10 RNA-Seq reads, others are not shown).

The assembly of the variation site in *A. thaliana* was supported by WGS reads. First, we found five WGS reads of *A. thaliana* crossing the deletion site in *A. thaliana*: SRR960120.29259367, SRR960120.21081404, SRR960120.5972951, SRR960120.5719552 and SRR960120.5381474. In addition, we found 10 *A. thaliana* WGS reads that matched the regions flanking the target variation site (1 kb at each side) in *A. lyrata* (SRR960120.13297623, SRR960120.12170852, SRR960120.9521381, SRR960120.24626408, SRR960120.10501244, SRR960120.5719552, SRR960120.5381474, SRR960120.11832388, SRR960120.24996622 and SRR960120.3443345) but no WGS reads of *A. thaliana* that matched the region of *A. lyrata* that are corresponding to the deletion of *A. thaliana* in position, which confirmed the deletion in *A. thaliana.*

# AT5G60790

*A. thaliana* TTGAAGCTACTGACATGTCTTCCCTGGAGGCTGTTGTTAGCTGTGATGAGGAGAGGTTGAGGTTAGAGAAAGAAGTTGAAATTCTTGTAC

*A. lyrata*  TTGAAGCTACTGACATGTCCTCCCTTGAGGCTGTTGTTAGCTGTGATGAAGAGAGGTTGAGGTTGGAGAAGGAAGTTGAAATTCTTGTTG

*C. rubella*  TTGAAGCCACTGACATGTCTTCCCTTGAGGCTGTTGTTAGCTGTGATGAGGAGAGGTTGAGGTTGGAGAAGGAAGTTGAGATTCTTGTTG

*A. thaliana* AGCAGG-------------------------------------------------------------------------------ATGAT

*A. lyrata*  AGCAGgtttgtgtcaatcaatactagattggattctaaattgagctttatattcgtgcttgatgcttattatggttgtttttatcagGAT

*C. rubella*  AGCAGgtttgtgtaattcaacaatagtctagatggttgattgaactttgttttcttgttggattctaatcatggttatttccagGATGAT

*A. thaliana* GGAGGCGGAGAGCGTCTTCAATCTATCTATGAGAGGTTAGACGCCATGGATGCAGAAACCGCTGAGAAACGTGCTGCTGAAATTCTGTTT

*A. lyrata*  GCTGGTGGCGAGCGTCTTGAATCTATCTATGAGAGGTTAGAAGCCATGGATGCTGCAACCGCTGAGAAGCGTGCTGCTGAAATTCTGTTT

*C. rubella*  GGAGGCGGAGAGCGTCTTGATTCTATATATGAGAGGTTGGAAGCCATGGATGCTGCAACTGCTGAGAAGCGTGCTGCTGAAATTTTGTTT

The *A. thaliana* gene *AT5G60790* lost most of an internal intronic region, and it simultaneously added a 1-bp-long bp segment to an upstream exon and a 2-bp-long segment of a downstream exon. This deletion did not cause any frame shift events. The active expression of the gene is supported by SRR360152.27511987, SRR360152.5551754, SRR360152.3714364, SRR360152.20330546, SRR360152.5802329, SRR360152.18989859, SRR360152.22319328, SRR360152.12356508, SRR360152.32568059, and SRR360152.17396096 (>10 RNA-Seq reads, others are not shown).

The successful splicing of the target region of *A. lyrata* is supported by SRR800644.217146, SRR800645.1122384, SRR800644.1915568, SRR800644.1107995, SRR800644.4032156, SRR800644.3655120, SRR800644.878290, SRR800644.3726605, SRR800645.4387793, and SRR800644.6479510 (>10 RNA-Seq reads, others are not shown).

The successful splicing of the target region of *C. rubella* is supported by SRR797558.12339013, SRR797558.12313361, SRR797555.44261608, SRR797558.15912182, SRR797558.7828538, SRR797556.19963521, SRR797557.14596202, SRR797557.29671557, SRR797555.35989397, and SRR797557.9873097 (>10 RNA-Seq reads, others are not shown).

The assembly of the variation site in *A. thaliana* was supported by WGS reads. First, we found seven WGS reads of *A. thaliana* crossing the deletion site in *A. thaliana*: SRR960120.9045295, SRR960120.8117772, SRR960120.13390819, SRR960120.22183963, SRR960120.12816472, SRR960120.11279963 and SRR960120.16689361. In addition, we found >10 *A. thaliana* WGS reads that matched the regions flanking the target variation site (1 kb at each side) in *A. lyrata* (SRR960120.4842189, SRR960120.2330477, SRR960120.15562867, SRR960120.1036350, SRR960120.13845969, SRR960120.8117772, SRR960120.20778119, SRR960120.11279963, SRR960120.27656924 and SRR960120.7242852, others are not shown) but no WGS reads of *A. thaliana* that matched the region of *A. lyrata* that are corresponding to the deletion of *A. thaliana* in position, which confirmed the deletion in *A. thaliana.*

1. Phytozome. http://www.phytozome.net/.

2. Long Q, Rabanal FA, Meng D, Huber CD, Farlow A, Platzer A et al. Massive genomic variation and strong selection in *Arabidopsis thaliana* lines from Sweden. Nat Genet. 2013;45(8):884-90.

3. Gregor Mendel Institute of Molecular Plant Biology. http://downloads.gmi.oeaw.ac.at/downloads/nordborg/.

4. The Sequence Read Archive of NCBI. http://www.ncbi.nlm.nih.gov/sra/.

5. Yang YF, Zhu T, Niu DK. Association of intron loss with high mutation rate in *Arabidopsis*: implications for genome size evolution. Genome Biol Evol. 2013;5(4):723-33.
